# Probing Self-Diffusion of Guest Molecules in a Covalent Organic Framework: Simulation and Experiment

**DOI:** 10.1021/acsnano.3c12167

**Published:** 2024-06-11

**Authors:** Lars Grunenberg, Christopher Keßler, Tiong Wei Teh, Robin Schuldt, Fabian Heck, Johannes Kästner, Joachim Groß, Niels Hansen, Bettina V. Lotsch

**Affiliations:** † Max Planck Institute for Solid State Research, Heisenbergstr. 1, Stuttgart 70569, Germany; ‡ Department of Chemistry, Ludwig-Maximilians-Universität (LMU), Butenandtstr. 5-13, Munich 81377, Germany; § Institute of Thermodynamics and Thermal Process Engineering, University of Stuttgart, Pfaffenwaldring 9, Stuttgart 70569, Germany; ∥ Institute for Theoretical Chemistry, University of Stuttgart, Pfaffenwaldring 55, Stuttgart 70569, Germany; ⊥ E-conversion, Lichtenbergstrasse 4a, Garching 85748, Germany

**Keywords:** pulsed field gradient NMR, diffusion, covalent organic framework, molecular dynamics, grand canonical Monte Carlo

## Abstract

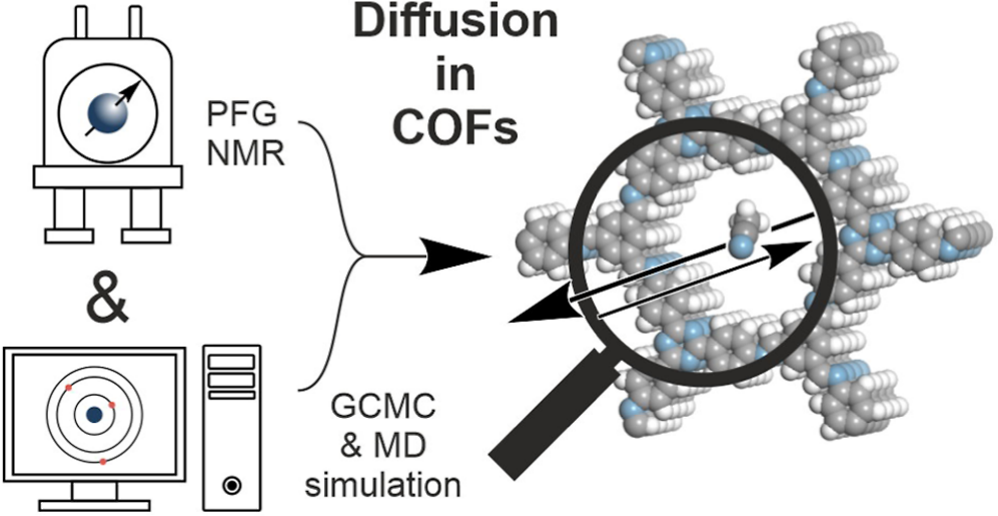

Covalent organic frameworks (COFs) are a class of porous materials whose sorption properties have so far been studied primarily by physisorption. Quantifying the self-diffusion of guest molecules inside their nanometer-sized pores allows for a better understanding of confinement effects or transport limitations and is thus essential for various applications ranging from molecular separation to catalysis. Using a combination of pulsed field gradient nuclear magnetic resonance measurements and molecular dynamics simulations, we have studied the self-diffusion of acetonitrile and chloroform in the 1D pore channels of two imine-linked COFs (PI-3-COF) with different levels of crystallinity and porosity. The higher crystallinity and porosity sample exhibited anisotropic diffusion for MeCN parallel to the pore direction, with a diffusion coefficient of *D*_par_ = 6.1(3) × 10^–10^ m^2^ s^–1^ at 300 K, indicating 1D transport and a 7.4-fold reduction in self-diffusion compared to the bulk liquid. This finding aligns with molecular dynamics simulations predicting 5.4-fold reduction, assuming an offset-stacked COF layer arrangement. In the low-porosity sample, more frequent diffusion barriers result in isotropic, yet significantly reduced diffusivities (*D*_B_ = 1.4(1) × 10^–11^ m^2^ s^–1^). Diffusion coefficients for chloroform at 300 K in the pores of the high- (*D*_par_ = 1.1(2) × 10^–10^ m^2^ s^–1^) and low-porosity (*D*_B_ = 4.5(1) × 10^–12^ m^2^ s^–1^) samples reproduce these trends. Our multimodal study thus highlights the significant influence of real structure effects such as stacking faults and grain boundaries on the long-range diffusivity of molecular guest species while suggesting efficient intracrystalline transport at short diffusion times.

## Introduction

Two-dimensional (2D) covalent organic frameworks (COFs) are a class of materials that combine a high level of tunability with intrinsic structural porosity on a crystalline, covalently linked polymeric backbone. Their chemical structure can be tuned with atomic precision, rendering these materials an attractive scaffold for diverse applications, including gas storage and separation, sensing, electrochemical energy storage, and heterogeneous (photo)catalysis.^1−9^

The typically large specific surface areas of these materials, and in particular the spatial arrangement of building blocks as encoded in the shape of the pore channels featuring adjustable diameters in the nanometer range, enable the utilization of confinement effects in heterogeneous catalysis, similar to those well-known from enzymes as biological catalysts.^10^ Spatial confinement in these pores allows a precise arrangement and relative orientation of catalytic centers and substrates in the pore channels and modulates the local concentration of reactants in the cavities.^11,12^ These effects can be used as a handle to tailor product selectivity in catalytic reactions, e.g., by suppressing oligomerization in l-lactide synthesis from lactic acid.^13^ Recently, Emmerling et al. demonstrated that the ordered structural porosity of COFs enhances selectivity for (mono)macrocyclization during a ruthenium-catalyzed olefin metathesis reaction, favoring ring closing over oligomerization.^14^ While the variation of pore size forms the basis for these effects, interactions between the reactants as well as other molecules in the reaction mixture with the pore wall become more dominant with a reduction in pore size.^15^ Acid/base interactions between catalytic substrates and reaction intermediates can affect the reaction rate, while collision events with the pore walls alter the in- and outflow of reactants.^16,17^ This can lead either to localized concentration gradients affecting selectivity or, in the limiting case for very small pore diameters that exclude (competing) molecules entirely, enable further areas of application, such as molecular sieving or nanofiltration.^18−22^

Computer simulations are well established in the field of porous media. Molecular dynamics (MD) simulations are a versatile tool to study self- and collective diffusion not only in crystalline porous media such as zeolites, MOFs, and COFs^23^ or carbon nanotubes^24^ but also in complex amorphous materials if a reasonable structural model is available.^25^ In contrast to zeolites^26−30^ and MOFs,^31−34^ the investigation of self-diffusion in COFs so far focused on light gases such as hydrogen, nitrogen, carbon dioxide, methane, and ethane.^35−39^ Molecular simulations in conjunction with experimental investigations have led to a fundamental understanding of nanoconfinement effects,^40^ but such combined studies so far exclusively focused on MOFs^41^ and zeolites.^42^ Therefore, we herein present a combined experimental and computational study of the self-diffusion of acetonitrile and chloroform in the 2D covalent organic network PI-3-COF using pulsed field gradient nuclear magnetic resonance (PFG-NMR) spectroscopy and grand canonical Monte Carlo (GCMC) and MD simulations. Using two samples with identical composition but differences in their real structure effects (i.e., crystallinity and porosity), we demonstrate the influence of pore confinement on the diffusivities of molecular probes.

## Results and Discussion

### Synthesis and Characterization of COFs

**Figure 1:**
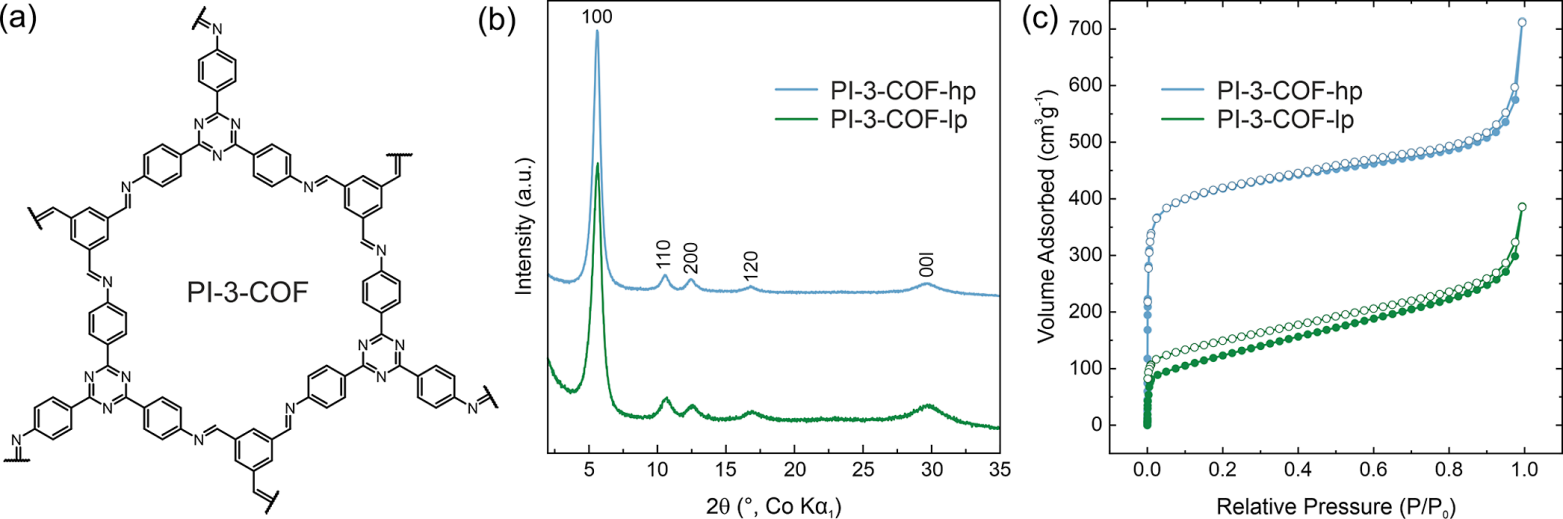
(a) Chemical structure of a single pore of PI-3-COF. (b) XRPD pattern and (c) N_2_ adsorption isotherm comparison of PI-3-COF-lp (green) and PI-3-COF-hp (blue).

Imine-linked PI-3-COF has been synthesized from 1,3,5-triformyl benzene and 4,4′,4″-(1,3,5-triazine-2,4,6-triyl)trianiline under solvothermal conditions according to a previously reported procedure.^43^ We synthesized two samples of PI-3-COF, named PI-3-COF-lp (low porosity) and PI-3-COF-hp (high porosity) in the following, depending on the selected drying procedure (see Supporting Information for details). Fourier transform infrared (FT-IR) spectra of the yellow powdered materials indicate the successful condensation to the imine-linked frameworks, represented by the imine bond vibration (v_C=N_) at 1630 cm^–1^ (Figure S 2). The spectra for both samples appear essentially indistinguishable due to their identical chemical composition. Structural analysis by X-ray powder diffraction (XRPD) with Co–K_α1_ radiation shows four narrow reflections at 2θ = 6.6, 11.5, 13.2, and 17.8°, indexed as 100, 110, 200, and 120 reflections (space group *P*6̅), and a broad stacking reflection (00l) centered at 2θ ≈ 30° (Figures S 4 and S 5). Bragg peaks in the XRPD pattern (Figure 1b) appear essentially at identical positions for both hp- and lp-materials, but with a reduced full width at half-maximum (fwhm) for the hp sample, hinting at a better structural definition and long-range order, i.e. crystallinity, of PI-3-COF-hp compared to its lp derivative. 100, 110, and 200 reflections appear broader, with reduced intensity for PI-3-COF-lp (Figure S 5) and show a slight but visible shift [Δ(2θ) < 0.1°] to higher angles, reminiscent of, but less pronounced than, a reduction in in-plane coherence and contraction due to drying induced stress.^44^ A Pawley refinement (Figure S 4) thus gives slightly reduced unit cell parameters of *a* = *b* = 17.9 Å and *c* = 3.47 Å for PI-3-COF-lp, compared to *a* = *b* = 18.0 Å and *c* = 3.48 Å for PI-3-COF-lp. Scanning electron and transmission electron microscopy (SEM/TEM) images show agglomerated polycrystalline spherical particles and a polydisperse distribution of (secondary) particle sizes, approximately centered at ∼300 nm in diameter (Figures S 7 and S 8) for both samples. Some agglomerates show sizes of multiple μm. The surface of these particles is decorated with stings, consisting of crystallites with average diameters of a few tens of nanometers (Figures S 9 and S 10). Nitrogen gas sorption experiments (Figure 1c) show a limited nitrogen uptake for PI-3-COF-lp and reveal BET surface areas of *S*_BET_ = 442 and 1620 m^2^ g^–1^ and total pore volumes of 0.60 and 1.1 cm^3^ g^–1^ for PI-3-COF-lp and hp, respectively (Figures S 11 and S 12). Calculated pore size distributions by quenched solid density functional theory (QSDFT) based on a carbon model for cylindrical pores are centered at 1.7 nm for both PI-3-COF-lp and hp (Figures S 11b and S 12b). With respect to the characterization data shown, we find that the difference between lp and hp lies in the extent of crystallinity, i.e., structural definition of the two samples, caused, for example, by inaccessible pores or disorder in PI-3-COF-lp and is not attributed to a difference in chemical composition. This leads to a reduced porosity in the case of PI-3-COF-lp compared to that in PI-3-COF-hp. Based on these findings, we envisaged using these samples as a basis for PFG-NMR diffusion experiments to investigate the effect of real structure effects such as crystallinity and porosity on the diffusivity.

### Probing Diffusion Experimentally by PFG-NMR

Due to its abundant use as a solvent in general organic and COF synthesis,^45^ acetonitrile (MeCN) was selected as a proxy to probe the self-diffusion of molecular reactants or intermediates in the pore system of a COF. In particular, MeCN loaded into the pores of PI-3-COFs showed sufficiently long T_2_ relaxation times for PFG-NMR experiments (s.b., Table S 1) compared to other probe molecules (Table S 3). Excess amounts of liquid on the outer particle surface or in the interparticle space distort the diffusion experiment and result in a major signal in the ^1^H NMR spectrum with bulk liquid-like mobility. To allow for more selective filling of the pores in the materials, we exposed the vacuum-dried materials to saturated acetonitrile vapor in air. Consequently, condensation of acetonitrile into the pores of the material occurred. As indicated by a single, broadened, and downfield-shifted signal for acetonitrile due to confinement,^46^ which is centered at δ = 3.7 ppm in the ^1^H NMR spectrum compared to the narrow signal for the isolated liquid at δ = 1.9 ppm (Figure S 18), the liquid mainly condensed into the pores, instead of interparticle voids which would yield signals closer to the free liquid. The mass of the samples increased after this solvent vapor treatment, corresponding to loadings of 25 wt % (PI-3-COF-lp) and 39 wt % (PI-3-COF-hp) of acetonitrile, respectively. Despite these high loadings, the appearance of the loaded materials was identical to the (dry) pristine materials. No liquid was visible on the surface.

PFG-NMR is a useful, nondestructive spectroscopic technique capable of tracking molecular motion and transport on a broad range of distances, varying from nanometers to hundreds of micrometers. By probing NMR signal attenuations at different diffusion times (Δ), spatial decoding of different diffusion regions and thus localized as well as long-range information on the structure of porous materials can be obtained.^47,48^ Fitting of the NMR signal obtained by the PFG method using the Stejskal-Tanner^49^ equation (eq 1) yields the diffusion coefficient (*D*) as a function of the gradient field strength (*g*), the gradient pulse duration (δ), and the gyromagnetic ratio of the probed nuclei (γ).
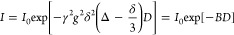
1To select appropriate gradient strengths and observation parameters, the relaxation times of the molecules under study are required.^50^ Both longitudinal relaxation times (T_1,lp_ = 1.8 s) and transverse relaxation times (T_2,lp_ = 0.54 ms) of acetonitrile loaded onto PI-3-COFs were between 1 and 3 orders of magnitude shorter compared to the bulk liquid at 300 K (Table S 1), diagnostic of smaller molecular mobility within the pores.^51^ On the one hand, this observation provides further evidence that MeCN is primarily located in the pores of the material, while, on the other hand, short spin–spin relaxation times (T_2_) lead to a fast decay of signal intensity in NMR experiments. This sets an experimental upper limit for the gradient pulse duration δ, as well as the diffusion time Δ. Long pulse durations and observation times lead to a bad signal-to-noise ratio because most of the signal has decayed due to relaxation^50^ before the signal can be measured. At the same time, uniform and stable gradients in the spectrometer require a technically limited minimum duration for the gradient pulse, setting the lower limits for δ during the PFG experiment.^47^

**Figure 2:**
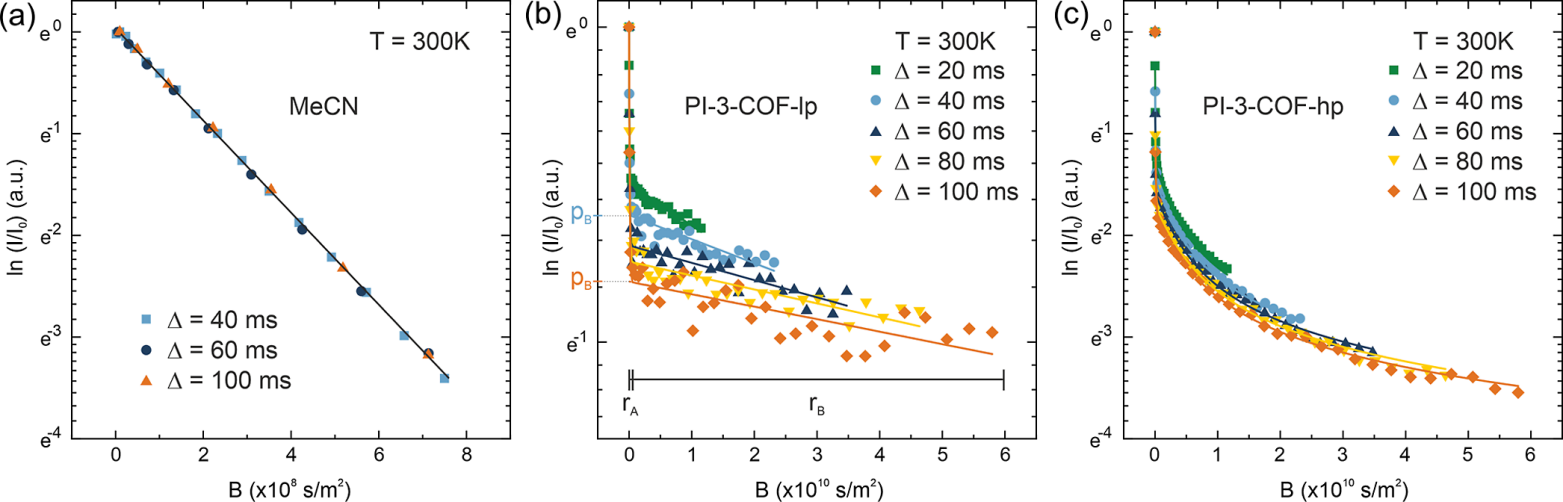
PFG-NMR spin–echo attenuation for (a) liquid MeCN, (b) MeCN-loaded PI-3-COF-lp, (c) MeCN-loaded PI-3-COF-hp with varying diffusion times (Δ) at *T* = 300 K. Lines represent fits with a mono- or biexponential model for MeCN and MeCN-loaded COFs, respectively.

For the presented PFG experiments, we chose the minimum technically possible value with our spectrometer of δ = 0.3 ms (at high gradients *g*_max_ = 900 Gs^1^ cm^–1^) to acquire PFG spin–echoes for diffusion times Δ = 20–100 ms using a stimulated echo (ste) pulse sequence (see Supporting Information for details). As shown in Figure 2, the spin–echo attenuations appear nonlinear for both samples, although linearity in the semilogarithmic representation would be expected for regular isotropic diffusion as observed for bulk acetonitrile (Figures 2a and S 16). The course of the signals can be separated into two regimes: a steeply decreasing initial range for small gradients (*r*_A_) and then a slowly decaying range toward large gradients (*r*_B_) at a fixed pulse duration δ = 0.3 ms. This behavior is characteristic of a distribution of diffusion coefficients, for example, observed in porous materials^52^ including zeolites^53,54^ and MOFs,^55^ where regions with different translational mobilities are found. This behavior can be observed in these materials for example for molecules diffusing inside versus outside of crystallites.^56^ A biexponential model (eq 2) can fit attenuations with this behavior, where *p*_i_ reflects the population of the region (*i*) with diffusivity *D*_i_.^52,57^ The diffusivities of both regions appear as linear ranges in the semilogarithmic plot.
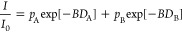
2The observed signal attenuations in PI-3-COF-lp (Figure 2b) are in good agreement with this simple biexponential model. For varying diffusion times, different slopes are visible in the range *r*_B_ toward high gradients (Figure 2b), indicating a dependence of the diffusivity *D*_B_ on diffusion time Δ. With variation of Δ, also the population *p*_i_, which can be interpreted as the *y*-intercept of the linear, slowly decaying intensity extrapolated to *B* = 0 (Figure 2b), changes. The population *p*_B_ decreases with longer observation times (Figure 2b). This phenomenon indicates a molecular exchange between both regions in the material, expected for open pore channels in PI-3-COF, and can be used by the NMR tracer exchange method^58^ to determine the fraction of molecules and their mean lifetime τ_i_ within these regions.^59,60^ With a defined macroscopic particle geometry, e.g., in single crystals, or for spherical particles, their diffusivity and average lifetime allow to estimate mean particle/crystallite sizes.^55,61,62^ Unfortunately, the wide distribution of particle sizes and shapes in our materials, as observed by electron microscopy (Figures S 7 and S 8), does not allow for this analysis. Applying the simple biexponential model to PI-3-COF-lp yields two diffusion coefficients of *D*_A_ = 1.7(2) × 10^–8^ m^2^ s^–1^ and *D*_B_ = 1.4(1) × 10^–11^ m^2^ s^–1^, with Δ = 20 ms at *T* = 300 K. A comparison of the exchange behavior between these regions at reduced temperature down to *T* = 280 K shows that the population *p*_A_ drops at reduced temperatures, and the exchange between both regions becomes less prominent (Figure S 21). Analysis of the population *p*_B_ at temperatures between *T* = 300 K and *T* = 280 K as a function of Δ further corroborates this finding, evident from a slower decrease of *p*_B_ vs Δ at reduced temperature (Figure S 23). The diffusivity *D*_A_ = 1.7(1) × 10^–8^ m^2^ s^–1^ exceeds the self-diffusion coefficient of pure acetonitrile (*D*_s_ = 4.5(1) × 10^–9^ m^2^ s^–1^, Figure S 17) at *T* = 300 K by 1 order of magnitude. The high diffusivity and strong temperature dependence of *p*_B_ (Figure S 23) suggest that *D*_A_ corresponds to an averaged diffusion of liquid acetonitrile molecules, which exchanged with the gas phase during the time of the NMR experiment.^63,64^ With reduced temperature, the vapor pressure of acetonitrile and thus the partial pressure of MeCN in the gas phase, as well as the probability for a phase exchange during the observation time, are reduced. Contrary to *p*_A_, we conclude that the molecules of the population *p*_B_ have not exchanged with the gas phase during the time of the NMR experiment and can be labeled as the fraction of molecules remaining within the particle. Their diffusivity *D*_B_ thus denotes intraparticle diffusion of acetonitrile within the pore channels of the polycrystalline particles of PI-3-COF. A comparison of *D*_B_ at constant temperature (Figure S 24) shows a decrease of *D*_B_ with increasing diffusion times Δ. In contrast to this, diffusion in the nonconfined, isotropic bulk liquid is independent of Δ (Figures 2a and S 16). Similar to observations in lithium-ion conductors^65^ and polycrystalline faujasite crystals,^66^ long-range diffusion of MeCN in PI-3-COF-lp, corresponding to long diffusion times Δ, is limited by transport barriers (e.g., grain boundaries or surface effects^53,67^), whereas at small displacements, these defects have less effect on the diffusion coefficient.^53^ To solely observe intracrystalline diffusion and reduce the influence of intercrystallite or interparticle diffusion resistances, *D*_B_ should ideally be measured at short diffusion times, where the mean square displacements (⟨*z*^2^⟩ ≈ 2*D*Δ) for most diffusing molecules in this time interval are smaller than the average crystallite diameter. However, due to technical limitations, Δ cannot be chosen arbitrarily small for high gradient values.^47^ Because the accessible isotropic diffusion lengths in the presented materials (μm range, Table S 2) exceed the observed crystallite size of a few tens of nanometers by SEM/TEM analysis (s. a.), only effective long-range diffusion coefficients can be obtained from the experiments. To estimate the order of magnitude for short-range diffusion in the pores of PI-3-COF, we extrapolated the experimental values for *D*_B_ toward short diffusion times in a phenomenological log(D)-log(Δ) plot, which has been used to describe, for example, restricted diffusion in zeolites (Figure S 25).^53^ The extrapolation suggests that *D*_B_ may approach values up to the order of 10^–10^ m^2^ s^–1^ for PI-3-COF-lp. The extrapolated values, however, should be interpreted with care as they might overestimate short-range diffusivity: the experimental diffusion coefficients for *D*_B_ may still contain a contribution of a fraction of acetonitrile diffusing in textural mesopores or small voids between individual crystallites, which would give rise to more bulk-like diffusivities. The presence of such textural pores can be inferred from acetonitrile vapor sorption experiments with PI-3-COF-lp (Figure S 13): the vapor sorption isotherm at 300 K shows a steep uptake at low relative pressures (*P*/*P*_sat_ < 0.13), corresponding to the filling of micropores, i.e., pore channels (structural pores). Toward higher relative pressure, a further but less steep uptake with pronounced hysteresis is visible. This uptake is attributed to the filling of textural mesopores. In turn, we conclude that some signal intensity during the NMR experiments may be caused by acetonitrile molecules in small textural pores, besides those in structural pores (pore channels of PI-3-COF). In addition, the linearity of the signal corresponding to intraparticle mobility *D*_B_ (Figure 2b) as well as the absence of additional signals during relaxation experiments suggests that the obtained PFG attenuation is not amenable to further quantitative differentiation of structural and textural pores. Relaxation times (Table S 1) as well as diffusion coefficients of molecules in these different pore regimes may appear superimposed and thus indistinguishable, likely influenced by a fast exchange between them relative to the NMR experiment time scale.

The PFG-NMR signal attenuation curves for MeCN-loaded PI-3-COF-hp (Figure 2c) similarly show two separated ranges and exchanges between the corresponding regions, evident from an offset of the slowly decaying range (*r*_B_). In contrast to PI-3-COF-lp, however, the signal attenuation appears to be nonlinear. This phenomenon indicates a contribution of anisotropic diffusion, which is in line with a diffusion along the 1D pore channels of PI-3-COF. Similar attenuations have been observed for anisotropic diffusion in pore channels of aluminum fumarate MOFs^56,68^ and mesoporous silica SBA-15.^69^ To address this effect of anisotropy on the observed PFG signal attenuation for MeCN in PI-3-COF-hp, the second term of the simple biexponential model was adjusted to a previously developed anisotropic model for hierarchically porous SBA-15 catalysts (eq 3), considering one-dimensional diffusion (channels) in particles, which are randomly oriented in space.^47,70^ Notably, this model uses a simplified approximation to account for the molecular exchange between the different regions (*p*_A_ and *p*_aniso_) in the material, based on the more complex solution developed by Splith et al.,^56^ which requires a negligibly small population *p*_A_ of the region with isotropic diffusion. Similar to the assumptions for the hierarchically ordered SBA-15 materials, however, we need to consider a fraction of MeCN present in small textural pores at the respective loadings of MeCN in the PI-3-COF samples under study, which is in contrast to the boundary conditions of the model by Splith et al.
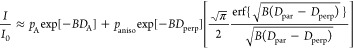
3Applying eq 3 to the observed signal attenuations for PI-3-COF-hp gives an isotropic diffusion coefficient *D*_A_ and two different anisotropic diffusion coefficients for movement of molecules parallel (*D*_par_) and perpendicular (*D*_perp_) to the channel direction. The fit shows excellent agreement with experimental data (Figure 2c) and yields two diffusion coefficients of *D*_A_ = 2.2(1) × 10^–8^ m^2^ s^–1^ and *D*_par_ = 6.1(4) × 10^–10^ m^2^ s^–1^, with Δ = 20 ms at *T* = 300 K and *D*_perp_ → 0, which is in line with the structural model of PI-3-COF consisting of closely stacked 2D layers that restrict diffusion between the layers, e.g., perpendicular to the channel direction. Analogous to the exchange behavior observed for PI-3-COF-lp, the material shows temperature-dependent molecular exchange between both regions, with *D*_A_ comprising contributions of gas diffusion through gas–liquid exchange during the observation time (Figure S 22). Extrapolation of the experimental values for *D*_par_ in the log(D)-log(Δ) plot^53^ gives values up to the order of 10^–9^ m^2^ s^–1^, similar to diffusion in the bulk liquid, toward short diffusion times. In summary, diffusion in the high porosity sample is less affected by defects or limited pore accessibility, resulting in an observable anisotropic diffusion parallel to the channel direction that is on average 1 to 2 orders of magnitude faster compared to PI-3-COF-lp.

To further complement the findings with a different solvent molecule, we assessed relaxation times of other probe molecules (Table S 3) adsorbed by PI-3-COF. Similar to acetonitrile, all solvents are characterized by short T_2_ relaxation in the μs range, with the shortest values for cyclohexane (T_2_ = 0.45 ms) and 1,4-dioxane (T_2_ = 0.31 ms), rendering them essentially unsuitable for diffusion studies by PFG-NMR. Despite the still fast T_2_ relaxation time (T_2_ = 0.55 ms), we obtained diffusion coefficients of *D*_par_ = 1.1(2) × 10^–10^ m^2^ s^–1^ (PI-3-COF-hp) and *D*_B_ = 4.5(1) × 10^–12^ m^2^ s^–1^ (lp) with Δ = 20 ms at 300 K for chloroform as a complementary diffusion probe (Figure S 28). While in the same order of magnitude, the values are smaller compared to diffusion coefficients of MeCN in these samples, in line with 1.7-fold reduced experimental self-diffusion of bulk chloroform (*D*_s_ = 2.6(1) × 10^–9^ m^2^ s^–1^) vs bulk MeCN (Figure S 27). Chloroform diffusion in PI-3-COFs shows generally the same qualitative trends as observed for MeCN, i.e., on average 1 to 2 orders of magnitude faster diffusion in the high-porosity sample.

### Computational Modeling

**Figure 3:**
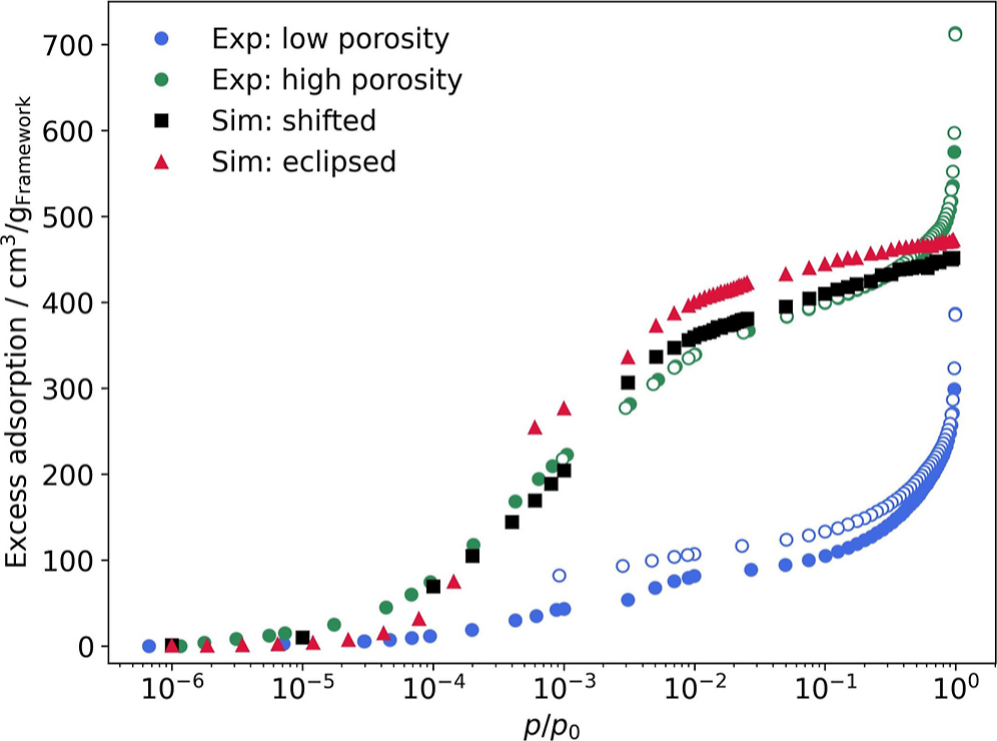
Comparison between experimental N_2_ adsorption isotherms at 77 K (low porosity in blue, high porosity in green) and simulated ones. The isotherm resulting from the shifted structure is depicted in black squares, the one from the eclipsed stacked structure in red triangles.

**Figure 4:**
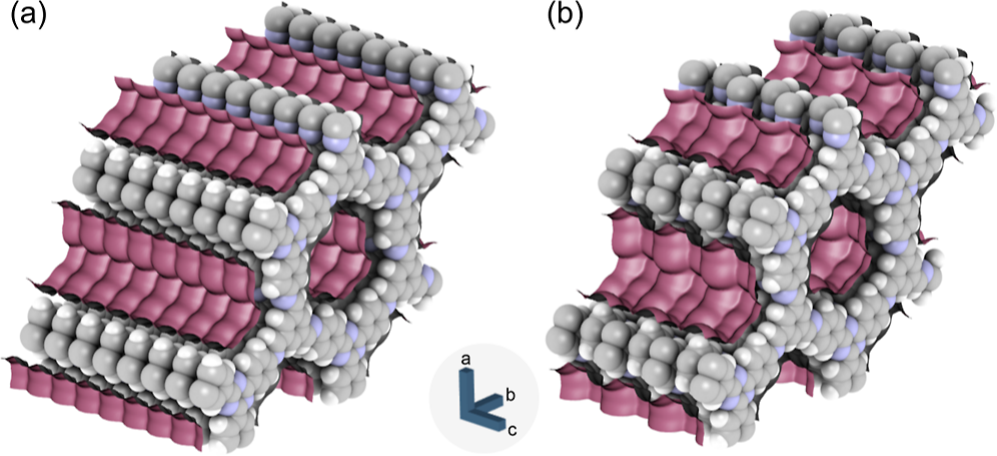
Visualization of the eclipsed (a) and the shifted structure (b) of PI 3-COF. Violet surfaces depict the N_2_ accessible pore surface based on van der Waals parameters.

Following previous work,^71^ the structural model obtained from XRPD experiments was refined by density functional calculations under periodic boundary conditions, as described in more detail in the Supporting Information. Figure 3 shows simulated and experimental excess nitrogen adsorption isotherms for PI-3-COF. The two experimental curves correspond to the lp and hp samples, while the two simulated curves correspond to model structures in which the layers are either perfectly eclipsed (red triangles), or where two adjacent layers are slightly shifted by approximately 1.7 Å in an alternating way (black squares). In the shifted model, the first and the third layer, the second and the fourth layer, and so on are eclipsed. This model mimics the effect of offset layer stacking, often found in COFs, for example, as layer displacement in randomized directions.^71−75^ In both cases, the interlayer distance was fixed to a value of 3.65 Å resulting from the DFT optimization for the shifted structure. The two structures are visualized in Figure 4. The good agreement between the simulated isotherms and the experimental curve of the hp sample indicates a high degree of crystallinity and accessibility of the experimental sample. In contrast to our previous work,^71^ no scaling factor was required to account for the finding that the simulation usually overestimated the experimental isotherm. The divergence of the experimental isotherms close to the saturation pressure results from condensation of nitrogen in textural macropores and is therefore not captured in the simulation, which is based on an infinite ideal structure. As found previously,^71^ the isotherm corresponding to the shifted structure shows a smoother increase in loading with increasing pressure compared to the eclipsed structure. Despite rather small structural differences in the two model COFs, a qualitative difference between the two simulated isotherms is visible in the medium pressure range between *p*/*p*_0_ = 10^–4^ and 10^–2^. However, the good agreement between experiment and simulation for the artificial, idealized structural model over the entire pressure range is distinct. This finding suggests that the real structure of the material is characterized by small shifts between the different layers.

The self-diffusion coefficients of acetonitrile in the two structures at 300 K amount to 1.0(2) × 10^–9^ m^2^ s^–1^ in the perfectly eclipsed structure and to 0.7(1) × 10^–9^ m^2^ s^–1^ in the shifted structure. Compared to the bulk liquid value of 3.8(2) × 10^–9^ m^2^ s^–1^ at 298 K, predicted by the molecular model, these coefficients correspond to a reduction by a factor of 3.8 (eclipsed) and 5.4 (shifted). The bulk value is close to the experimentally determined self-diffusion coefficients reported in the literature (*D* ≈ 4.2 × 10^–9^ m^2^ s^–1^)^76,77^ and the present study (*D*_s_ = 4.5(1) × 10^–9^ m^2^ s^–1^). However, since the simulated value of bulk diffusion does not exactly match the experimental one, it is reasonable to compare the ratios of the bulk and pore diffusion coefficients in addition to the absolute values. The ratio between the diffusion coefficients of liquid MeCN in bulk (this study) vs PI-3-COF-hp (PFG) of 7.4 obtained in the present work is in good agreement with the ratio of 5.4 for the simulated bulk value vs the simulation result for the shifted structure at 300 K.

In other simulation works, ratios of 2.0 and 3.2 were reported for the diffusion of MeCN in a carbon nanotube of 1.5 nm diameter^78^ and an amorphous silica sample of 2.4 nm diameter,^79^ respectively. Experimental studies reporting the self-diffusion of acetonitrile under confinement show a more diverse picture. In pores smaller than 1 nm, self-diffusion coefficients on the order of 10^–11^ m^2^ s^–1^ were measured for zeolite NaX^59^ and porous carbon,^80^ respectively. In a sol–gel glass with a reported diameter of 2.9 nm, a diffusion coefficient of 1.1 × 10^–9^ m^2^ s^–1^ was obtained,^81^ i.e., similar to the diffusion coefficient reported for a mesoporous MCM-41 sample (pore size 3.6 nm, *D* = 9.9 × 10^–10^ m^2^ s^–1^)^82^ and larger than the diffusion coefficient reported for a porous carbon (pore size 4.8 nm, *D* = 6.0 × 10^–10^ m^2^ s^–1^).^80^ Experiments probing a pore size similar to the one in the present work are scarce. For a MCM-41 sample with a pore size of 2 nm, a diffusion coefficient of 2.7 × 10^–10^ m^2^ s^–1^ was reported,^82^ which is relatively close to the value obtained for the hp sample in the present work. We note that the self-diffusion coefficient under confinement is affected not only by the pore diameter, but also by interactions of the diffusing compound with the pore wall, in particular in narrow pores. Therefore, the comparison with other materials can only provide a qualitative picture.

For chloroform, simulated self-diffusion coefficients of 0.3(1) × 10^–9^ and 0.2(1) × 10^–9^ m^2^ s^–1^ were obtained in the perfectly eclipsed and in the shifted structures, respectively. Compared to the simulated bulk liquid value of 2.3(1) × 10^–9^ m^2^ s^–1^ at 298 K, predicted by the molecular model, these coefficients correspond to a reduction by a factor of 7.7 (eclipsed) and 11.5 (shifted). The bulk value is close to the experimentally determined self-diffusion coefficient of 2.6(1) × 10^–9^ m^2^ s^–1^ (300 K). The simulated diffusion coefficient for the shifted structure of 0.2(1) × 10^–9^ m^2^ s^–1^ is close to the experimentally determined for PI-3-COF-hp *D*_par_ = 1.1(2) × 10^–10^ m^2^ s^–1^ (PI-3-COF-hp), which is in line with the observations for MeCN as the diffusing molecule.

### Experiment vs Simulation

The present work aims at clarifying the comparability of the self-diffusion coefficient of acetonitrile in a COF obtained from MD simulations and PFG-NMR measurements. For this purpose, two model structures were investigated in conjunction with a fluid model that captures the bulk diffusion coefficient reasonably well. The theoretical model, applied to ideal structural models of isolated pore channels, suggests a comparably fast diffusion within the structural pore channels of PI-3-COF in both fully eclipsed and offset-stacked cases, albeit with slightly reduced diffusivity in the offset-stacked case. The obtained simulated diffusion coefficients are slightly lower, yet roughly of the same order of magnitude as isotropic diffusion in the bulk liquid. We complemented our simulation studies with experimental data obtained by PFG-NMR experiments. We observed short T_2_ relaxation times for the confined liquid in the pores of PI-3-COF. This limits diffusion times and pulse durations applicable during PFG experiments. Due to the limited crystallite sizes in the polycrystalline COF particles, the experimentally observed diffusion coefficients were limited to mid-to-long-range diffusion processes across multiple crystallites, given the technical limits for short pulse durations at high gradient strengths. From the obtained NMR signal attenuation, we identified multicomponent diffusion with open pore channels allowing the equilibrium exchange of molecules between the surrounding vapor phase and pore liquid. PFG measurements at reduced temperatures helped to assign these contributions by limiting the gas–liquid exchange. The experimental diffusivity in PI-3-COF samples was obtained as effective diffusion coefficients and found to decrease for long diffusion times. This behavior points at real structure effects, e.g., defects and surface barriers at crystal boundaries within and between the particles.^66,83^ For a sample of PI-3-COF with lower porosity, these effects are more dominant compared to the sample with higher porosity, which led to the observation of effective diffusivities on the order of 10^–11^ and 10^–10^ m^2^ s^–1^ for lp and hp samples, respectively. Extrapolation of the obtained diffusivities toward short diffusion times, i.e., small mean square displacements, indicates that short-range diffusion may be 1 (hp) to 2 orders (lp) of magnitude faster than the observable long-range diffusion. The high-porosity sample of PI-3-COF showed anisotropy in diffusion, characterized by diffusivities that agree well with the simulated values for the offset-stacked model, both being in the order of 10^–10^ m^2^ s^–1^ for MeCN. In contrast, the reduced structural definition of the lp sample led to the observation of isotropic diffusion only. This observation hints at the dominant influence of diffusion barriers in the material, restricting the diffusion of acetonitrile to shorter displacements and reducing its mobility compared to the hp sample. Thus, we point out that limited structural order not only reduces the accessible pore volume, i.e., porosity, but also restricts the mobility of molecules via diffusion barriers. As these are essentially invisible to typical analytical techniques, including gas sorption experiments, PFG-NMR spectroscopy should be considered as a complementary method to assess diffusivity-dependent parameters, such as turnover frequency or selectivity of reactions with COFs as heterogeneous catalysts.

Comparing the experimental to the simulated results helps to pinpoint important insights into the real structure of the material. Our comparison between calculated nitrogen gas adsorption isotherms for eclipsed and offset structures in a pressure range *p*/*p*_0_ = 10^–4^ and 10^–2^ shows profound sensitivity for localized differences in the stacking and suggests small displacements of the layers in the material since the experimentally observed isotherm of PI-3-COF-hp closely resembles the simulated isotherms of the offset-stacked structure with appreciable agreement. Thus, simulated isotherms may serve as a handle to pinpoint local characteristics in the real structure of the material, although these simulations are generally based on artificial, idealized structural models.

## Conclusions

The combined experiments and simulations shine light on prevalent diffusion mechanisms and issues associated with the experimental determination of diffusion coefficients in COFs. However, the direct observation of pure short-range, i.e., undisturbed intracrystalline diffusion within the pore channels, requires large (ideally single crystalline) particles with pore channel lengths in the μm range. Most powdered COF materials obtained from typical synthetic procedures do not meet this requirement, and obtaining crystallite sizes in this range is a rarely tackled and challenging task for imine and other COFs.^84^ Nevertheless, our systematic computational and experimental study sets the stage for future exploration of diffusion processes in COFs and related systems. We propose that optimizing the synthesis conditions to obtain domain sizes in the μm range should be the basis for future studies. With these requirements in mind, we expect that the influence of pore sizes and the chemical structure of the pore walls as well as their surface polarity and the impact of meso-/macroporosities on diffusion processes become experimentally accessible.

## Methods

### Synthesis of PI-3-COF

PI-3-COFs with low porosity (-lp) and high porosity (-hp) were synthesized according to a previously described procedure.^43^ To a mixture of benzene-1,3,5-tricarbaldehyde (22.1 mg, 0.13 mmol, 1.0 equiv) and 4,4′,4″-(1,3,5-triazine-2,4,6-triyl)trianiline (46.8 mg, 0.13 mmol, 1.0 equiv) in mesitylene (2.7 mL) and 1,4-dioxane (1.3 mL), aqueous 6 M AcOH (0.5 mL) was added. The suspension was heated at 120 °C for 72 h. Suction filtration of the precipitate, washing with DMF (20 mL), THF (20 mL), and DCM (20 mL) and drying under reduced pressure, afforded PI-3-COF-lp (55.5 mg, 91%) as a yellow solid. PI-3-COF-hp (53.9 mg, 88%) was obtained with the same procedure extended by an additional Soxhlet-extraction of the material with MeOH and supercritical CO_2_ drying, instead of drying under reduced pressure.

### Vapor-Assisted Loading of COFs

PI-3-COFs (10–15 mg) loaded into a 2 mL vial were exposed to a vapor-saturated air atmosphere of acetonitrile or chloroform at 300 K for 2 h. The mass loading of solvent was determined on a balance. The sample was then quickly transferred to a 5 mm NMR tube and flame-sealed to limit evaporation of the adsorbed liquid. To allow for equilibration within the sample, the sealed tube was stored for at least 24 h prior to the NMR experiment.

### PFG-NMR Experiments

Diffusion measurements were performed in flame-sealed 5 mm NMR tubes on a Bruker AVANCE III 400 MHz spectrometer (*diff60* probe) with a stimulated-echo sequence^49^ (*diffSte* program, Bruker TopSpin) without sample spinning. Protons served as the observed nuclei. For a typical diffusion experiment with a COF, a gradient pulse with a duration of δ = 0.3 ms (*opt* shape), repetition times of 3–5 times T_1_, and diffusion times Δ = 20–100 ms were used. The gradient was varied linearly in 32 steps between 0 and 900 Gs/cm. Further details are described in the Supporting Information.

Diffusion experiments with bulk liquid (acetonitrile or chloroform) were performed in a tube-in-tube setup to reduce convection effects.^85^ A small diameter NMR tube filled with acetonitrile or chloroform was immersed in a 5 mm NMR tube with *d*-chloroform. In addition, the double stimulated-echo pulse (*dSte*) sequence was used to reduce convection effects on the diffusion experiment.^86^ A gradient pulse with a duration of δ = 1 ms (*opt* shape), repetition times of 3–5 times T_1_, and diffusion times Δ = 40; 60; 100 ms were used. The gradient was varied linearly in 16 steps between 0 and 75 Gs/cm.

## Data Availability

The data underlying this study, including additional material such as cif files, RASPA and Gromacs input files, and a Jupyter notebook containing the analysis, are available in the Data Repository of the University of Stuttgart (DaRUS) at 10.18419/darus-3269.

## Supporting Information Available

Discussions of methods and equipment used, synthetic procedures, FT-IR spectra, XRPD data and structure refinements, (pulsed field gradient) ^1^H NMR spectra, nitrogen gas sorption isotherms, pore-size distributions, BET plots, and acetonitrile vapor adsorption isotherms (PDF)

## Notes

An initial version of this manuscript has been previously submitted to a preprint server. Grunenberg, L.; Keßler, C.; Teh, T. W.; Schuldt, R.; Heck, F.; Kästner, J.; Groß, J.; Hansen, N.; Lotsch, B. V. Self-Diffusion of Acetonitrile in a Covalent Organic Framework: Simulation and Experiment. 2024. ChemRxiv. 10.26434/chemrxiv-2024-d9rmq (accessed May 13, 2024).
