# Supplementary material for: Probing Self-Diffusion of Guest Molecules in a Covalent Organic Framework: Simulation and Experiment
Source: ACS Nano. 2024 Jun 11;18(25):16091–100. doi: 10.1021/acsnano.3c12167 (PMC11210340; doi:10.1021/acsnano.3c12167)
Supplement: Supplementary file 1 — nn3c12167_si_001.pdf [file nn3c12167_si_001.pdf]

Supporting Information

Probing Self-Diffusion of Guest Molecules in a Covalent Organic Framework: Simulation and Experiment

Lars Grunenberg, [†,a,b] Christopher Keßler, [†,c] Tiong Wei Teh,[c] Robin Schuldt,[d] Fabian Heck,[a,b] Johannes Kästner,[d] Joachim Groß,[c] Niels Hansen.[c,*]and Bettina V. Lotsch.[a,b,e,*]

[a] Max Planck Institute for Solid State Research, Heisenbergstr. 1, 70569 Stuttgart, Germany

[b] Department of Chemistry, Ludwig-Maximilians-Universität (LMU), Butenandtstr. 5-13, 81377 Munich, Germany

[c] Institute of Thermodynamics and Thermal Process Engineering, University of Stuttgart, Pfaffenwaldring 9, 70569 Stuttgart, Germany

[d] Institute for Theoretical Chemistry, University of Stuttgart, Pfaffenwaldring 55, 70569 Stuttgart, Germany

[e] E-conversion, Lichtenbergstrasse 4a, 85748 Garching, Germany

† Authors contributed equally.

[*] Email: hansen@itt.uni-stuttgart.de and b.lotsch@fkf.mpg.de

Materials and Methods

General methods: All reactions, unless otherwise noted, were performed with magnetic stirring under inert gas (N_2 or Ar) atmosphere using standard Schlenk techniques. Reaction temperatures were electronically monitored as external heating block temperatures. Reagents were purchased from different commercial sources and used without further purification.

Infrared spectroscopy: IR spectra were recorded on a Perkin Elmer UATR Two FT-IR spectrometer equipped with an attenuated total reflection (ATR) measuring unit. IR data are reported in wavenumbers (cm^{-1}) of normalized absorption. The IR bands are characterized as w (weak), m (medium), s (strong), or br (broad).

Supercritical CO_2 activation: Activation of the methanol-soaked COF samples with supercritical CO_2 was performed on a Leica EM CPD300 critical point dryer.

Gas sorption measurements: Sorption measurements for COFs were performed on a Quantachrome Instruments Autosorb iQ MP with Nitrogen at 77 K. The samples were degassed for 12 h at 120 °C under vacuum prior to the gas adsorption studies. Pore size distribution was determined from nitrogen adsorption isotherms using the QSDFT cylindrical pores in carbon model for nitrogen at 77 K. For multipoint BET surface area calculations, pressure ranges were chosen with the help of the BET assistant in the ASiQwin software, which chooses BET tags in accordance with the ISO recommendations equal or below the maximum in grams per square meter.

Vapor sorption measurements: Vapor sorption experiments were performed on a Quantachrome Instruments Autosorb iQ MP with acetonitrile at different temperatures. The samples were degassed for 12 h at 120 °C under vacuum prior to the adsorption studies. Values of the adsorbed amount V_{STP} [cm^3g^{-1}] were converted to gravimetric adsorbed amount [g^1g^{-1}] = V_{STP}/22414*41.05.

X-ray powder diffraction (XRPD): X-ray powder diffraction experiments were performed on a Stoe Stadi P diffractometer (Co- $K\alpha_1$, Ge(111)) in Debye-Scherrer geometry. The samples were measured in sealed glass capillaries (OD = 0.7 mm) and spun for improved particle statistics.

Pawley refinements: Pawley refinements were performed using TOPAS v6. The background was corrected with Chebychev polynomials (Order 10). Simple axial and zero-error corrections were used together with additional corrections for Lorentzian crystallite size broadening.

Scanning electron microscopy (SEM): SEM SE (secondary electron) detector images were obtained on a Zeiss Merlin SEM.

Transmission electron microscopy (TEM): TEM analysis was performed with a Philips CM30 ST (300kV, LaB6 cathode). The samples were prepared dry onto a copper lacey carbon grid (Plano).

Computational methods: To simulate the self-diffusion-coefficient we use the following overall workflow. A COF structure is determined experimentally and then relaxed by Density Functional Theory (DFT) calculations. Adsorption loading is simulated in Grand Canonical Monte Carlo (GCMC) simulations. Molecular Dynamics (MD) simulation are then started with the results from GCMC simulations to obtain self-diffusion coefficients. The individual steps are now described in detail.

GCMC Adsorption: Starting from a COF structure determined by powder diffraction, we refined the framework using DFT calculations. Atomic partial charges of the framework are calculated on basis of a single point DFT calculation of the charge density of the framework applying the DDEC6 method.[1-4] The obtained structure is kept rigid during GCMC and MD simulations.

Adsorption isotherms were calculated by the GCMC methodology[5] implemented in RASPA molecular simulation package.[5] To describe adsorbate-adsorbate interactions we use the TraPPE force field for acetonitrile and nitrogen.[6, 7] Therein acetonitrile is modelled in a united-atom description containing 3 Lennard-Jones beads namely CH_3, C and N with atomic point charges on each. The quadrupole moment of nitrogen is taken into account by placing negative point charges on each nitrogen atom and a neutralizing charge in the center of mass of the molecule. For chloroform we use the molecular model proposed by Kamath *et al*.[8] Lennard-Jones parameters of the framework were taken from the DREIDING force field[9] and unlike interactions are calculated by Lorentz-Berthelot combining rules.[10, 11] The combination of TraPPE and DREIDING force fields has been

used extensively,[6, 7, 9] while for chloroform simulation studies in confinement are scarce. To compare to experiment we first determine the void fraction of the framework and then calculate excess adsorption. We use a spherical cut-off-radius of 14 Å for Van-der-Waals interactions and electrostatic interactions. Long range electrostatics are considered using Ewald summation.[12] Sufficient statistics are reached by 100,000 Monte Carlo cycles for equilibration and 100,000 to 200,000 cycles for production.. Experimental adsorption isotherms show excess adsorption. Therefore, the void fraction of the framework is obtained computationally. A detailed description is given in our earlier work.[13]

MD simulations were performed in Gromacs version 2019.6.[14, 15] The TraPPE force field model of acetonitrile is a linear one with fixed angles of 180° which may lead to problems in the force calculation for the bending potential. To cope with that the model is adjusted using the potential proposed by van der Spoel *et al*.[16]

We first evaluate the force field's ability to reproduce self-diffusion coefficients in the liquid phase. Good statistics are assured by running 10 independent simulations with starting velocities drawn from a Maxwell-Boltzmann distribution, 3 ns equilibration and 10 ns production with a timestep of 1 fs. Finite size effects are accounted by an extrapolation to an infinite box size. Therefore, we vary the size of the simulation box and the number of molecules accordingly. Temperature is kept constant by applying the Nose-Hoover-Thermostat.[17, 18] In NpT-simulations pressure is controlled by the Berendsen barostat.[19] Long range Van-der-Waals corrections and Ewald summation for electrostatics are used in liquid phases and simulation in pores. In bulk, we sample the Mean-squared displacement in xyz-direction from 1 to 5 ns and calculate the self-diffusion coefficient accord to the Einstein Equation

$$D_s = \frac{1}{2dN} \lim_{t \to \infty} \frac{\mathrm{d}}{\mathrm{d}t} \langle \sum_{i=1}^{N} |\boldsymbol{r}_i(t) - \boldsymbol{r}_i(0)|^2 \rangle \qquad \text{(Eq. S1)}$$

where $\boldsymbol{r}$ is the position of the center of mass of a molecule, N is the number of molecules and d is the dimension of the system. NpT-simulations and NVT-simulations with densities taken from NpT-simulations yield results within the margin of error.

To simulate the diffusion-coefficient in the COF pore we first insert a rigid COF pore into the simulation box. Adsorbate molecules are added until the previously determined average loading by GCMC calculations is reached. Then we follow the procedure

described for liquid simulations with following adjustments. In simulation a porous media acts as an external field which results in a change of the degrees of freedom of the simulated system.[20]

As in a 2D COF the diffusion process becomes one-dimensional after short timescales we sample only the mean squared displacement in direction of the pore axis, so the dimension in equation S1 reduces. Sampling the mean squared displacement in less dimensions reduces the quality of statistics. This is mitigated by extending simulation time to 5 ns equilibration and 20 ns production. We sample the MSD from 1 ns to 8 ns. Gromacs settings are ensured by a comparison to MD simulations in RASPA.

Solid-state nuclear magnetic resonance spectroscopy (ssNMR): Solid state NMR spectra (ssNMR) were recorded at room temperature in 4 mm ZrO_2 rotors on a Bruker Neo 600 MHz spectrometer (^{13}C: 150 MHz) using a Bruker BL4 triple resonance MAS probe. Standard instrument library pulse sequences were used. Chemical shifts were referenced relative to tetrakis(trimethylsilyl)silane (TTSS) (^{13}C, 3.51 ppm) measured as external standard.

Thermogravimetric analysis: Thermogravimetric analysis (TGA) was performed on a NETZSCH STA 449 F3 Jupiter. Measurements were carried out in Al_2O_3 crucibles with lids under synthetic air (20% O_2 in N_2) flow (10 mL/min) a temperature range between 25 and 500°C and a heating rate of 5 K/min. Baseline correction and buoyancy effects were compensated by subtracting reference measurements with an empty crucible.

Pulsed field gradient nuclear magnetic resonance spectroscopy (PFG-NMR): Diffusion measurements were performed in flame-sealed 5 mm NMR tubes on a Bruker Avance III 400 MHz spectrometer (*diff60* probe) between 270 and 300K with a stimulated-echo sequence[21] (*diffSte* program, Bruker TopSpin) without sample spinning. Protons served as the observed nuclei. A variable temperature control unit ensured constant sample temperature during the experiment. For temperatures below room temperature, a stream of chilled nitrogen was connected to the temperature control unit. To restrict the vapor filled volume to the temperature-controlled region below the sample spinner, a PTFE-silicone stopper was inserted into the NMR tube (see Figure S 1).

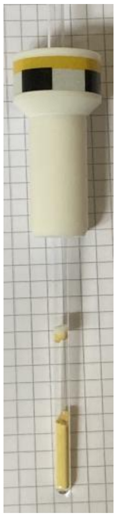

Figure S 1: NMR sample tube with a PTFE-silicone insert and PI-3-COF sample (yellow). The insert restricts the vapor volume of MeCN to the temperature controlled zone below the spinner.

Data processing was performed with the Bruker TopSpin 3.5 *Dynamics* module using automated peak picking, integration and fitting of the integrated signal *I* vs. gradient *B* according to the Stejskal-Tanner equation (Eq. S2), where the gradient factor B is defined by the gyromagnetic ratio (γ) of the observed nuclei, the gradient field strength (g), the gradient pulse duration (δ) and the diffusion time (Δ). For fitting of MeCN or acetonitrile in COFs modified bi-exponential versions of this equation were used, as described in the main text.

$$I = I_0 \; exp\left[-D\gamma^2 g^2 \delta^2 \left(\Delta - \frac{\delta}{3}\right)\right] = I_0 e^{[-DB]} \quad \text{(Eq. S2)}$$

T_1 relaxation times were determined using a standard inversion-recovery pulse sequence with a delay time d1 = 10 s. T_2 relaxation times were obtained by fitting the signal attenuation during a cpmg pulse sequence with a delay time d1 = 8 s. For a typical diffusion experiment with a COF, a gradient pulse with a duration of δ = 0.3 ms (*opt* shape), repetition times of 3 -5 times T_1, and diffusion times Δ = 20-100 ms were used. The gradient was varied linearly in 32 steps between 0 and 900 Gs/cm.

Diffusion experiments with pure, liquid acetonitrile or chloroform were performed in a tube-in-tube setup to reduce convection effects.[22] A small diameter NMR tube filled with acetonitrile or chloroform was immersed in a 5 mm NMR tube with *d*-chloroform. In addition, the double stimulated-echo pulse (*dSte*) sequence was used to reduce convection effects on the diffusion experiment.[23] A gradient pulse with a duration of

δ = 1 ms (*opt* shape), repetition times of 3 -5 times T_1 and diffusion times Δ = 40 ;60; 100 ms were used. The gradient was varied linearly in 16 steps between 0 and 75 Gs/cm.

Experimental Procedures

Synthesis of PI-3-COF: PI-3-COFs with low-porosity (-lp) and high-porosity (-hp) were synthesized according to a previously described procedure.[24] To a mixture of benzene-1,3,5-tricarbaldehyde (22.1 mg, 0.13 mmol, 1.0 eq.) and 4,4',4''-(1,3,5-triazine-2,4,6-triyl)trianiline (46.8 mg, 0.13 mmol, 1.0 eq.) in mesitylene (2.7 mL) and 1,4-dioxane (1.3 mL), aqueous 6 M AcOH (0.5 mL) was added. The suspension was heated at 120°C for 72 h. Suction filtration of the precipitate, washing with DMF (20 mL), THF (20 mL) and DCM (20 mL) and drying under reduced pressure, afforded PI-3-COF-lp (55.5 mg, 91%) as a yellow solid. PI-3-COF-hp (53.9 mg, 88%) was obtained with the same procedure extended by an additional Soxhlet-extraction of the material with MeOH and supercritical CO_2 drying, instead of drying under reduced pressure. **FT-IR** (ATR): v = 1579 (m), 1511 (vs), 1413 (m), 1369 (s), 1174 (w), 1141 (w), 1013 (w), 968 (w), 864 (w), 813 (m), 679 (w), 530 (w) cm^{-1}.

Supporting Analytical Data

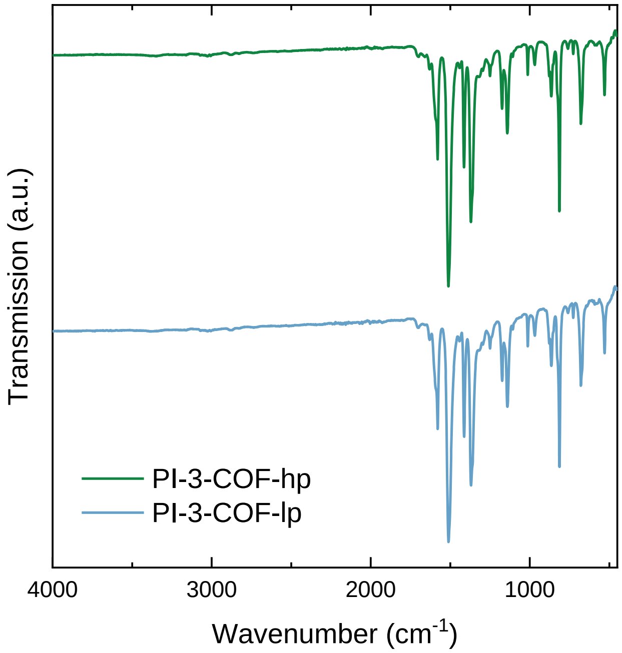

Figure S 2: FT-IR spectra of PI-3-COF-lp/-hp. Both spectra are essentially identical.

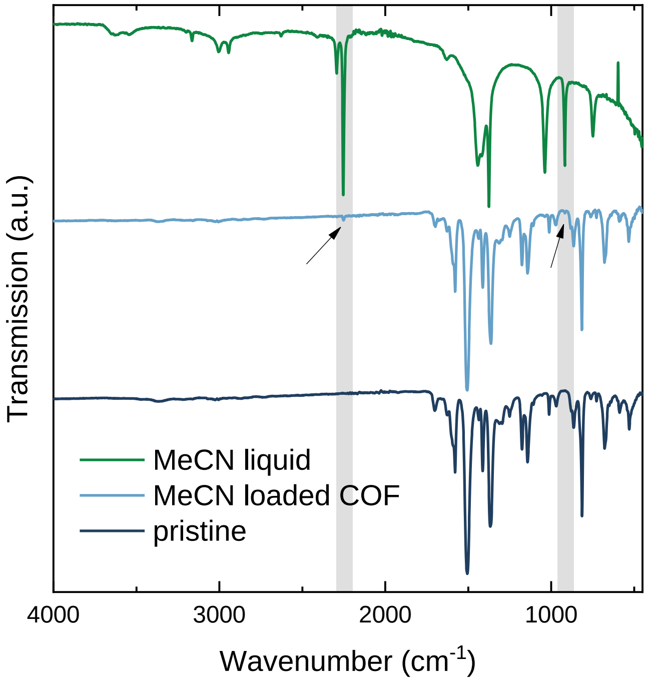

Figure S 3: FT-IR spectra comparison of liquid MeCN (green), pristine PI-3-COF-lp (dark blue) and PI-3-COF-lp loaded with MeCN (blue). Upon MeCN loading characteristic vibrations become visible, highlighted in gray.

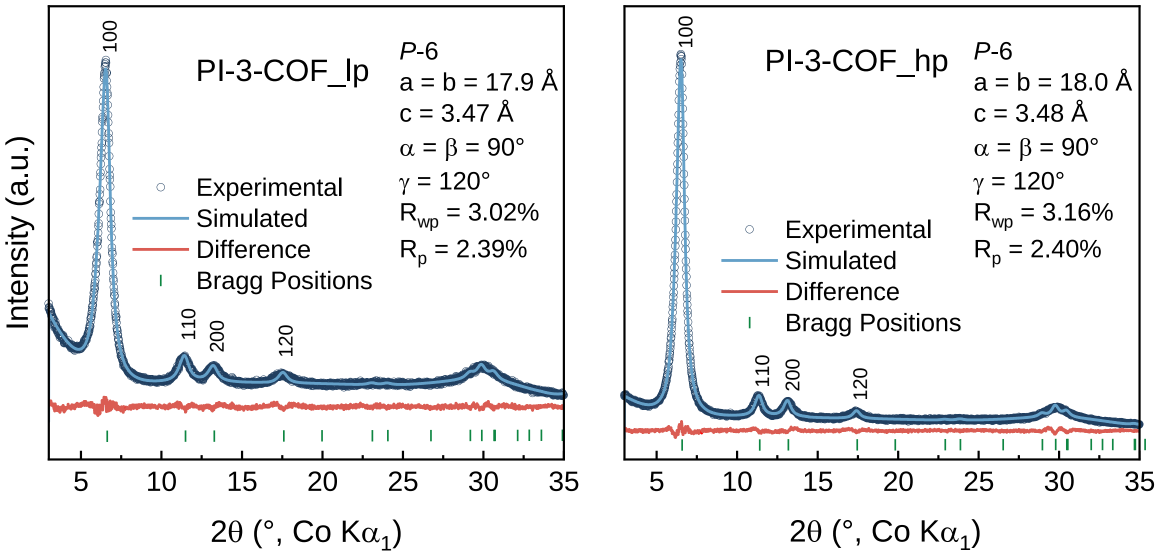

Figure S 4: Pawley refinements of XRPD data. Obtained cell parameters are in agreement with previous reports.

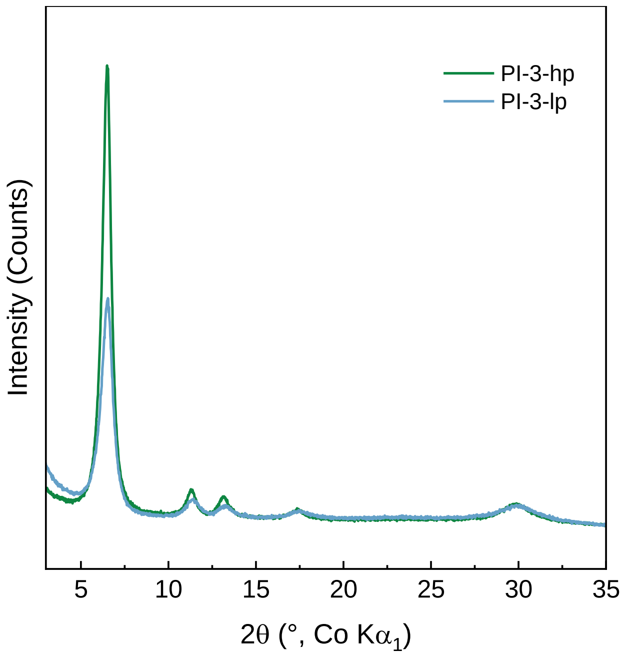

Figure S 5: XRPD comparison of PI-3-COF-lp/hp. PI-3-COF-hp shows higher crystallinity evident from better defined reflections (smaller FWHM), in agreement with its higher porosity.

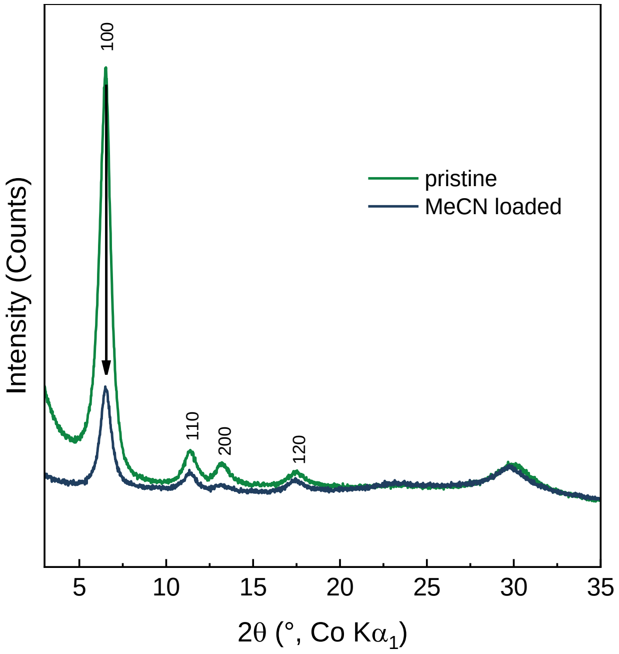

Figure S 6: XRPD comparison of pristine (green) and MeCN-loaded PI-3-COF-lp (blue). Intensities of 100/110/200 reflections decrease upon MeCN loading due to the reduced scattering contrast. This behavior was similarly described for water adsorption in COF pore channels.[25]

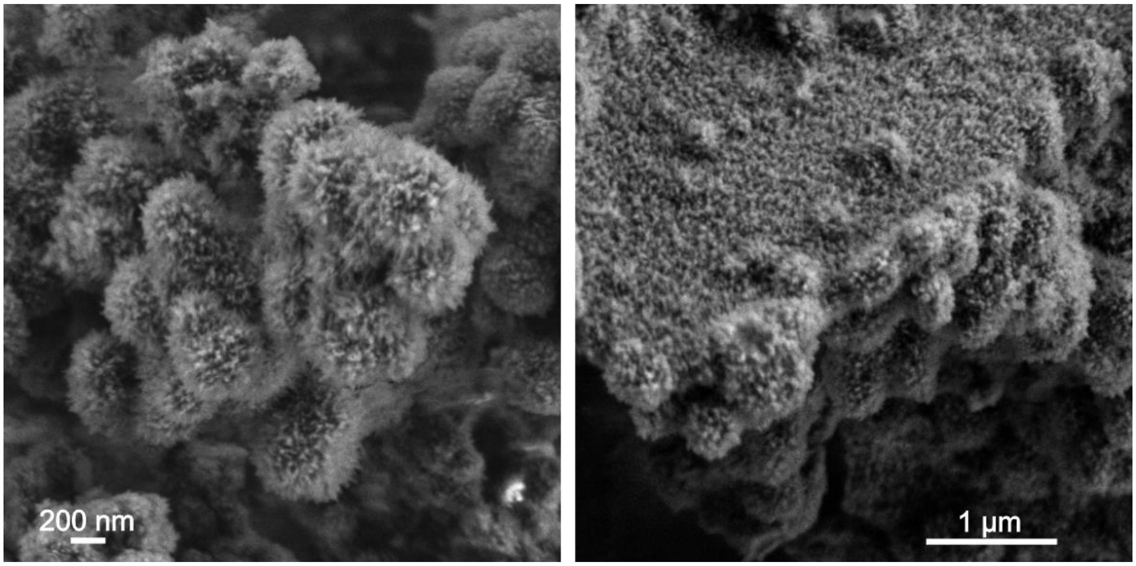

Figure S 7: SEM images of PI-3-COF-lp showing irregular spherical particles with a rough surface.

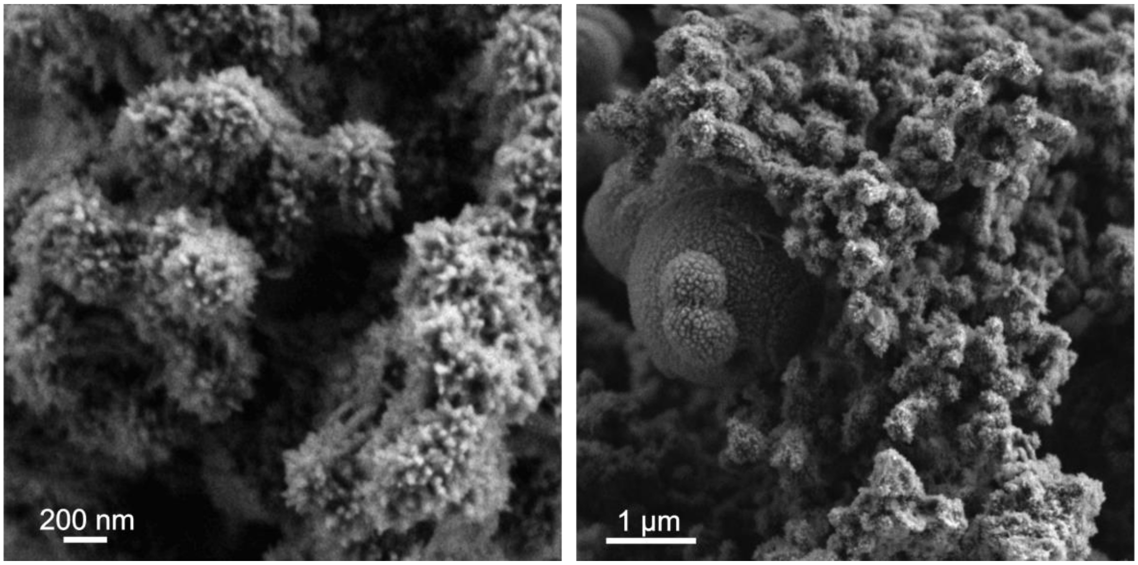

Figure S 8: SEM images of PI-3-COF-hp show agglomerated and intergrown irregular spherical particles similar to the lp with a rough surface.

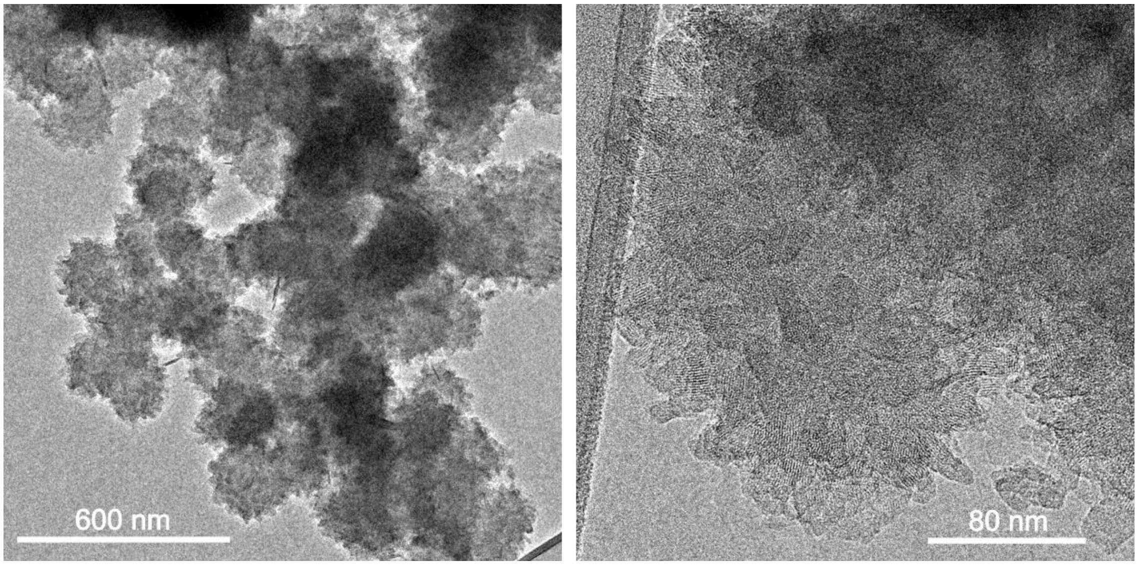

Figure S 9: TEM images of PI-3-COF-lp showing spherical particles with ~200 nm diameter and a rough surface with crystalline stings. The primary crystallite size is in the range of a few tens of nanometers.

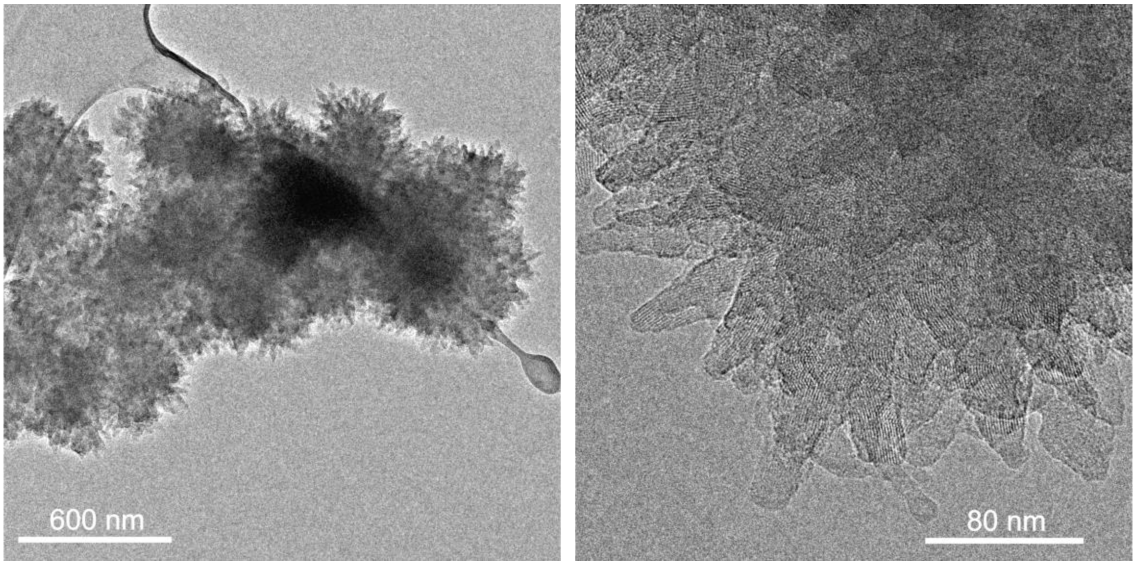

Figure S 10: TEM images of PI-3-COF-hp show similar morphology to PI-3-COF-lp with slightly larger spherical particles with a rough surface, decorated with crystalline stings.

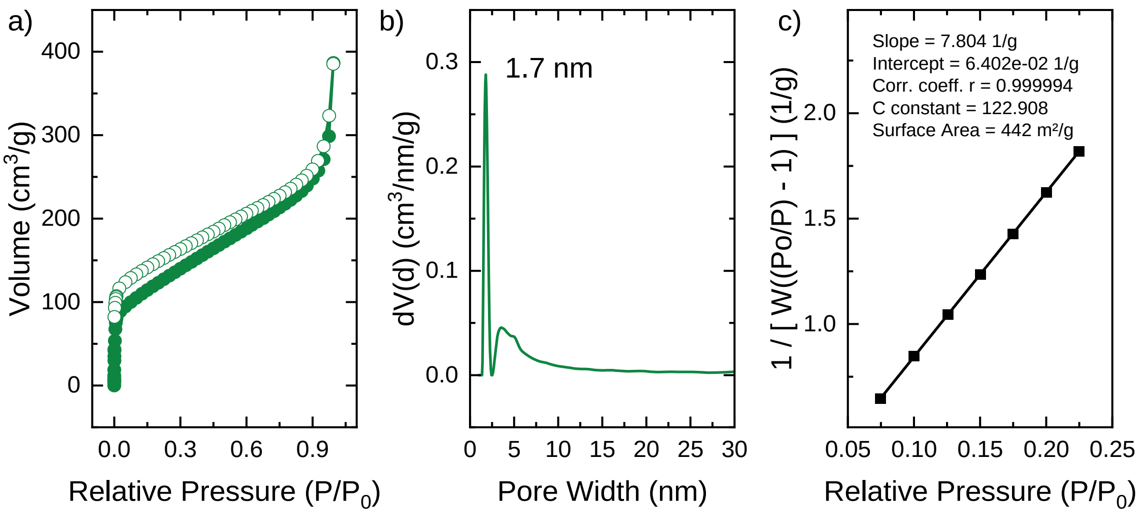

Figure S 11: N_2 sorption isotherm (a), pore-size distribution (b), and BET plot (c) for PI-3-COF-lp.

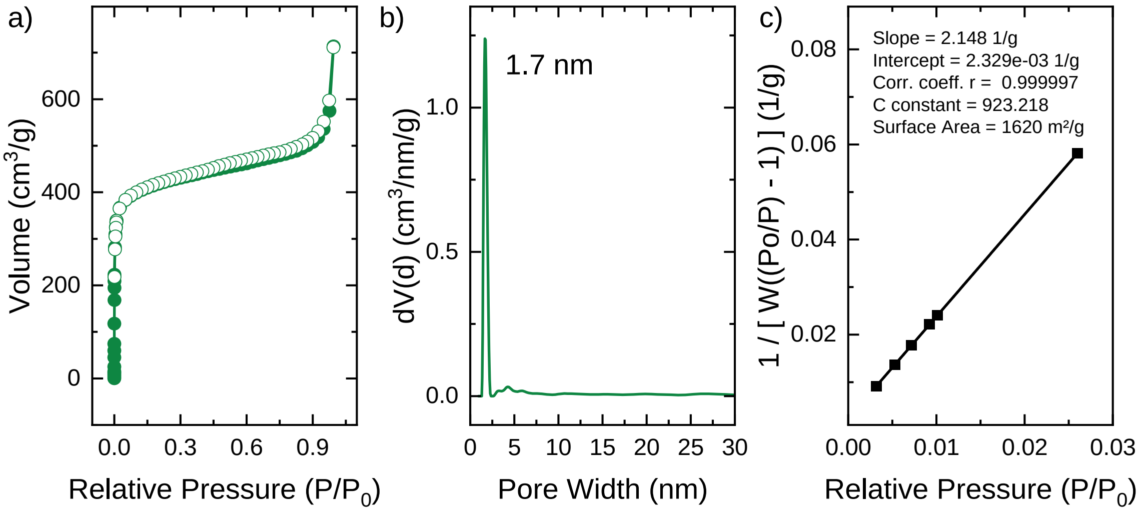

Figure S 12: N_2 sorption isotherm (a), pore-size distribution (b), and BET plot (c) for PI-3-COF-hp.

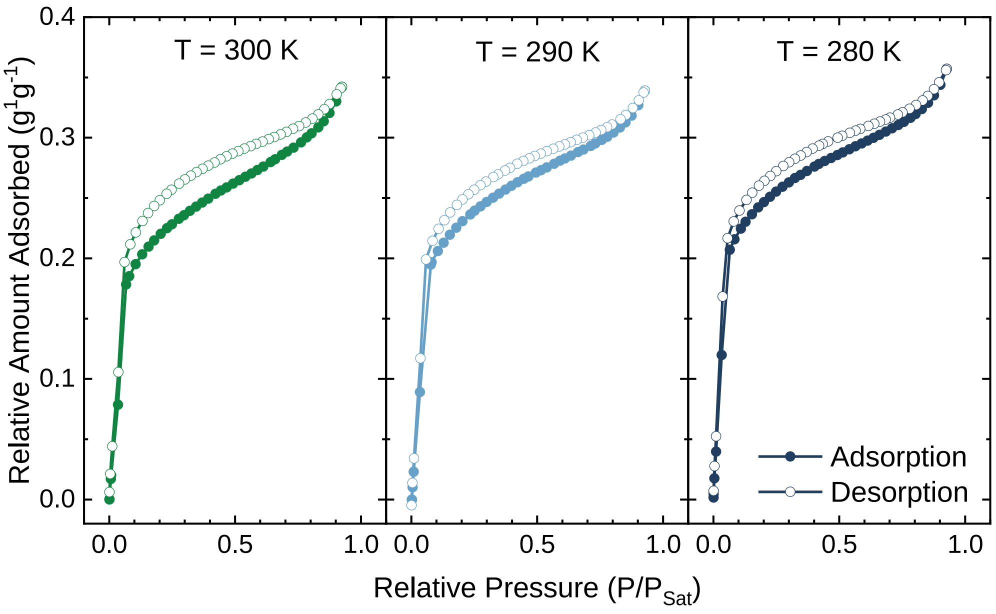

Figure S 13: MeCN vapor adsorption isotherm of PI-3-COF-lp at different temperatures. Filled dots represent data points of the adsorption branch, hollow dots those of the desorption branch, respectively.

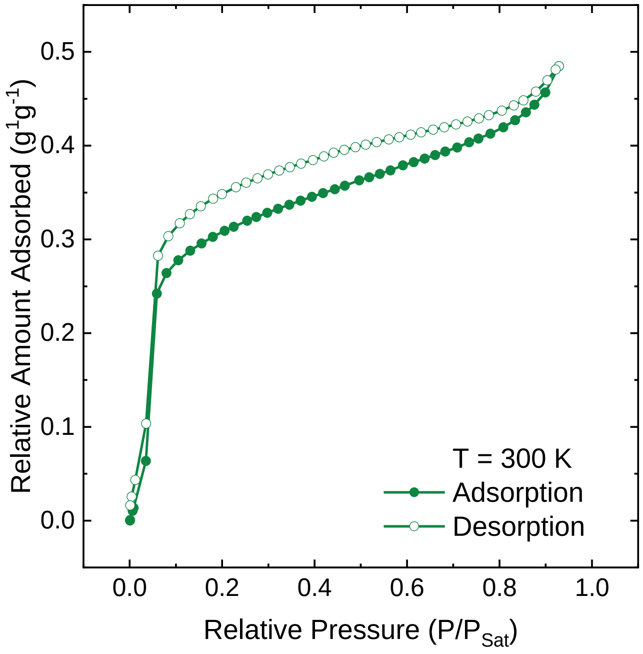

Figure S 14: MeCN vapor adsorption isotherm of PI-3-COF-hp at 300K. Filled dots represent data points of the adsorption branch, hollow dots those of the desorption branch, respectively.

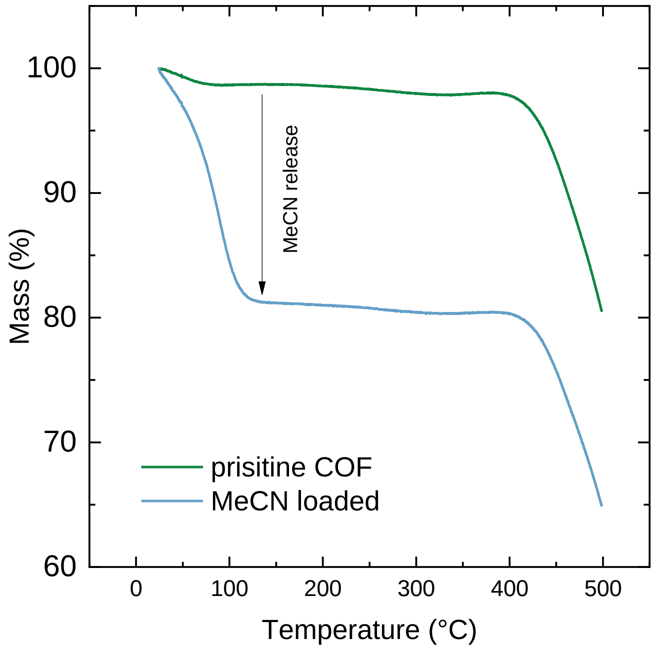

Figure S 15: Thermogravimetric trace of pristine (green) and MeCN-loaded (blue) PI-3-COF-lp under synthetic air flow. The mass loss <100°C (blue curve) is attributed to MeCN desorbed from the COF. Notably, the mass loss (~17%) may not be used to quantify the MeCN loading reliably, due to evaporation losses during TGA sample preparation.

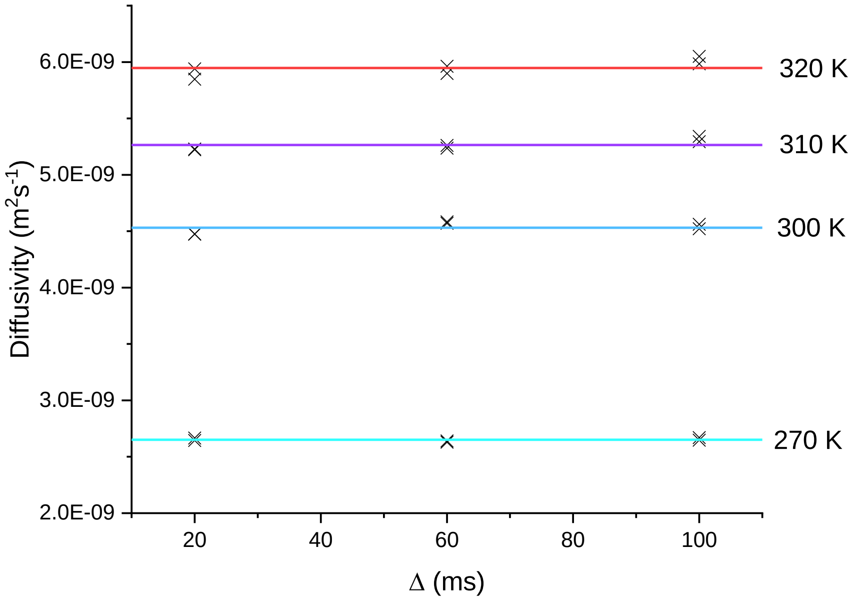

Figure S 16: Fitted diffusion coefficients from PFG NMR signal attenuations for acetonitrile at different temperatures. The diffusivity is independent of the diffusion time and increases for higher temperatures.

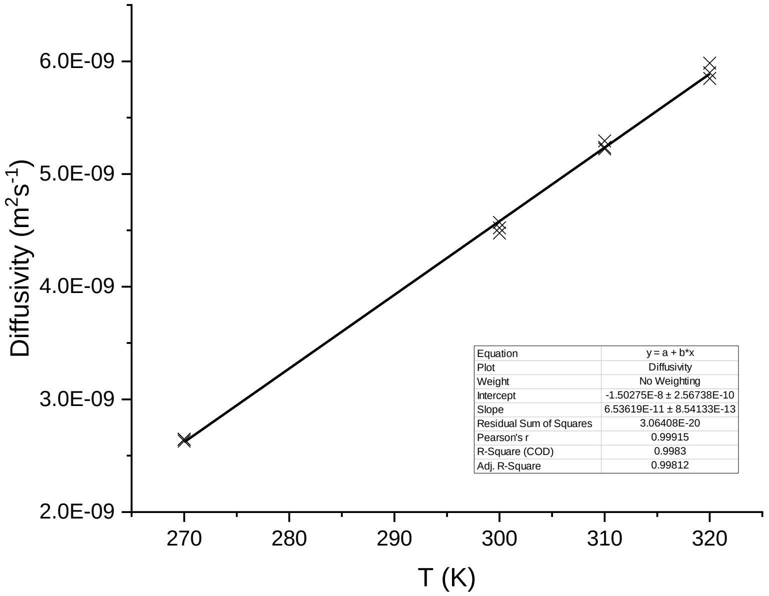

Figure S 17: Diffusion coefficients of acetonitrile plotted against temperature. As expected for regular, isotropic diffusion, the diffusivity shows a linear correlation to temperature, following the Stokes-Einstein equation. Diffusivities agree well with previous studies.[26-28] Notably, in this qualitative comparison the influence of viscosity changes is neglected.

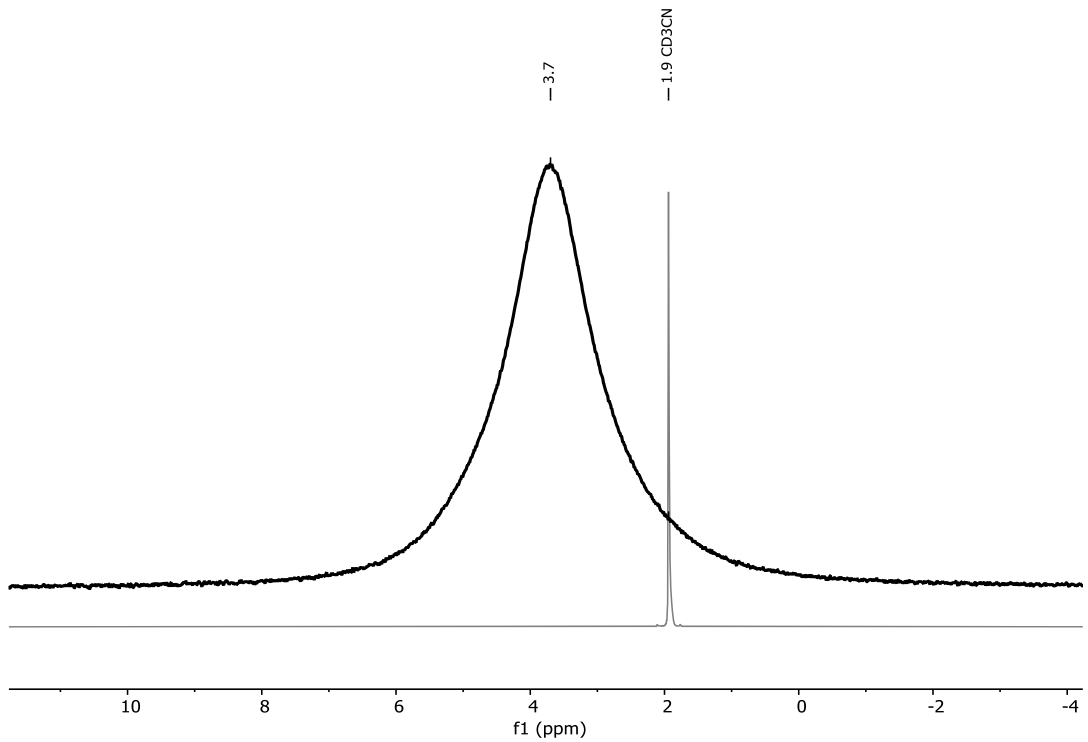

Figure S 18: ^{1}H-NMR spectrum of liquid acetonitrile (light grey, bulk) compared to the spectrum of acetonitrile loaded into the pores of PI-3-COF lp (black). Intensities are adjusted for visibility.

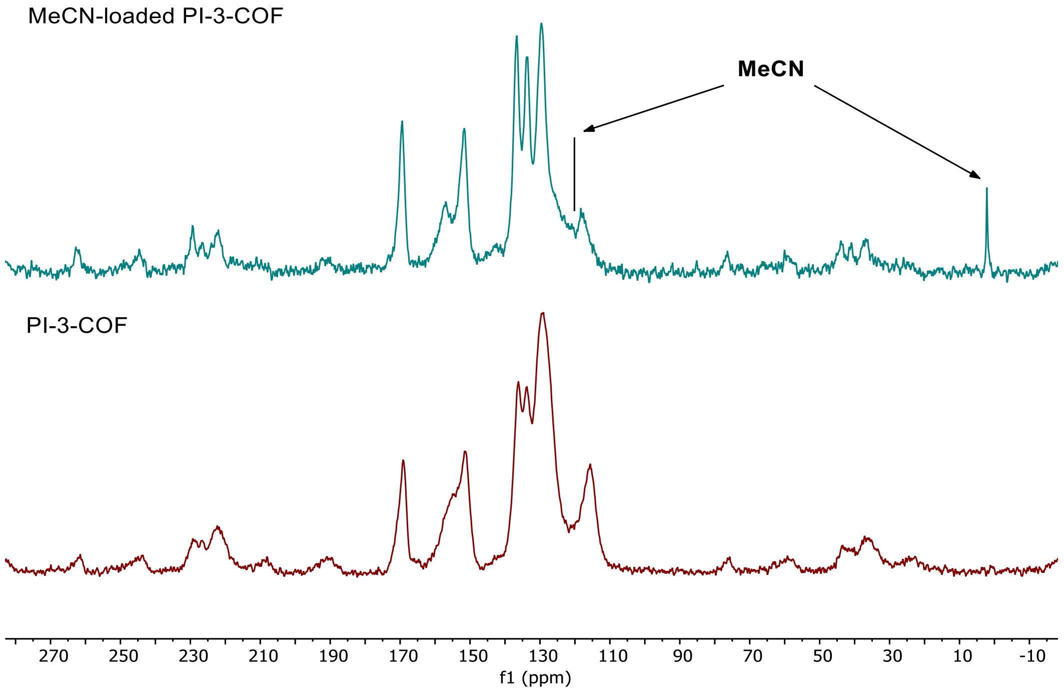

Figure S 19: ^{13}C-CP-MAS NMR spectrum (solid-state) of PI-3-COF-lp (red) and acetonitrile loaded PI-3-COF-lp (cyan). Note that the intensity of MeCN carbons cannot be quantified, due to cross polarization conditions.

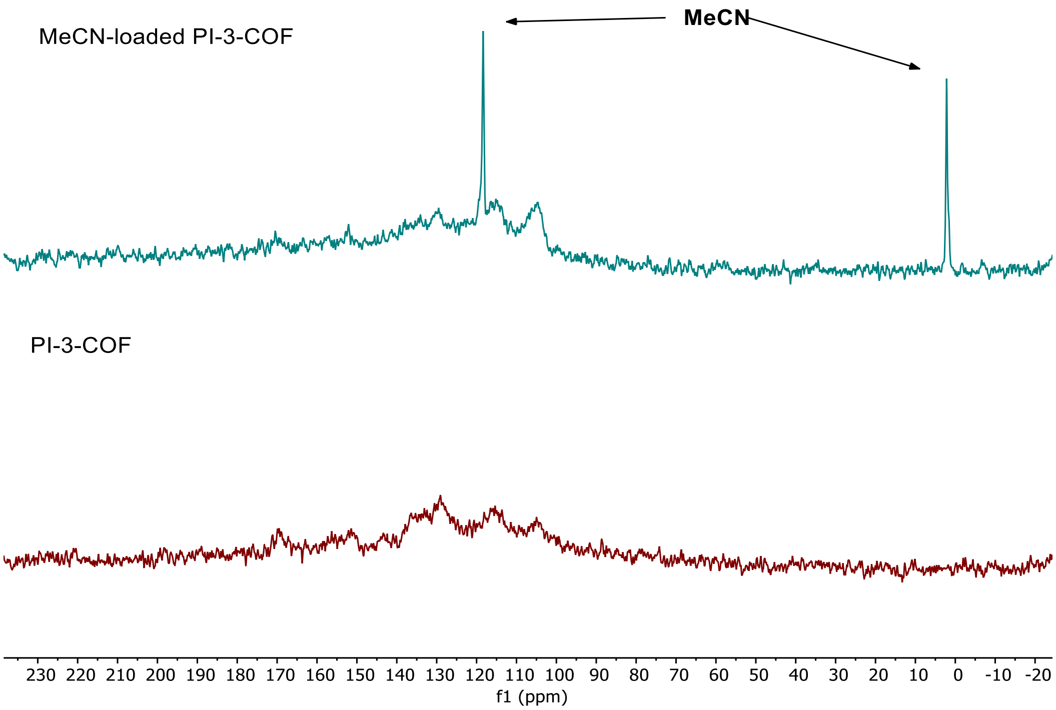

Figure S 20: ^{13}C-direct excitation MAS NMR spectrum (solid-state) of PI-3-COF-lp (red) and acetonitrile loaded PI-3-COF-lp (cyan). Here, intensities correlate to the number of nuclei, due to direct excitation of the carbon nuclei.

Table S 1: T_1/T_2 relaxation times of acetonitrile (MeCN) compared to acetonitrile loaded PI-3-COF-lp/-hp.

	T_1 [s]	T_2 [ms]
MeCN (T=300K)	14.2	850
PI-3 + MeCN (T=300K) -lp/-hp	1.80/1.94	0.54/0.85
PI-3 + MeCN (T=290K) -lp/-hp	1.62/1.81	0.49/0.77
PI-3 + MeCN (T=280K) -lp/-hp	1.41/1.66	0.45/0.70

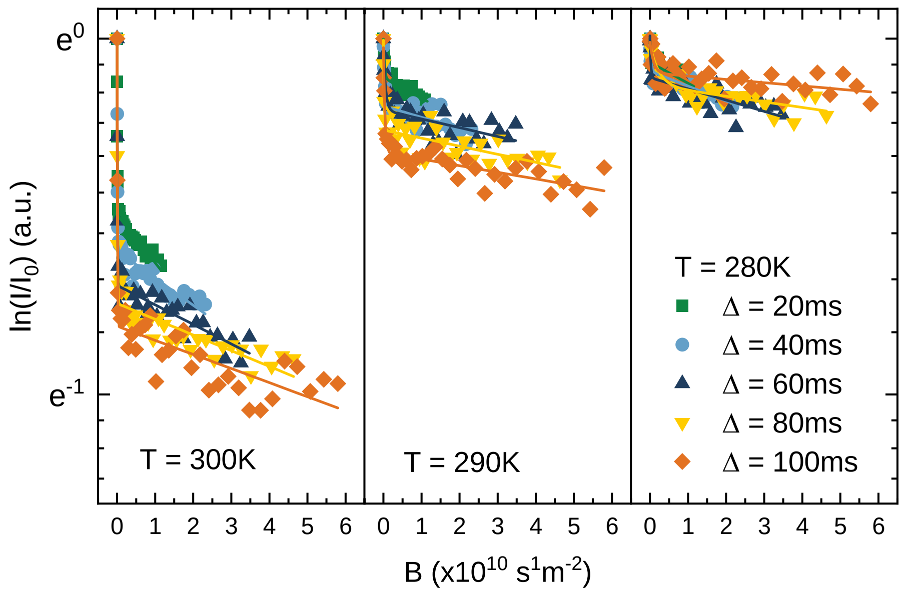

Figure S 21: PFG-NMR spin-echo attenuation for MeCN loaded PI-3-COF-lp with varying diffusion times (Δ) at different temperatures. Lines represent fits with a simple bi-exponential model.

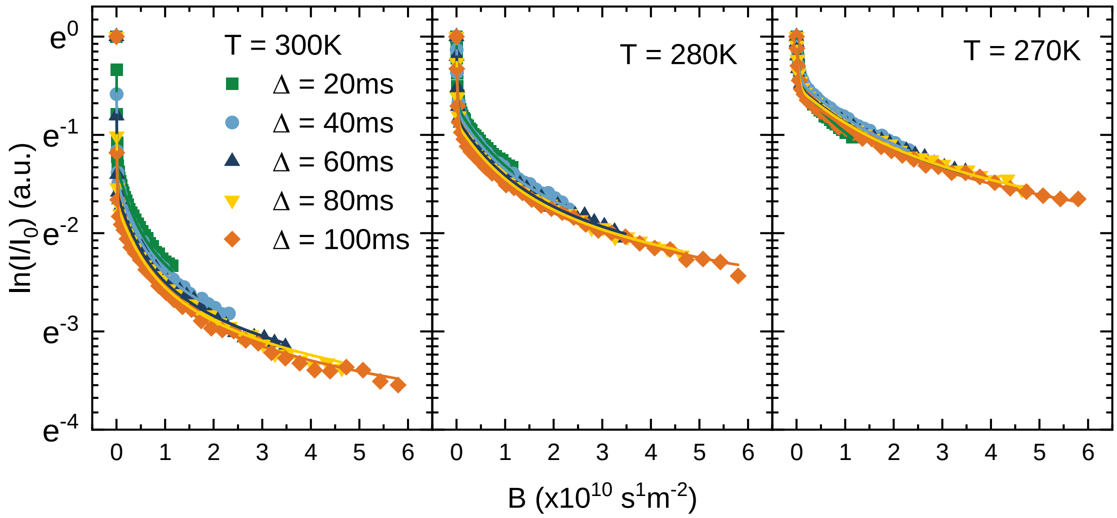

Figure S 22: PFG-NMR spin-echo attenuation for MeCN loaded PI-3-COF-hp with varying diffusion times (Δ) at different temperatures. Lines represent fits with an anisotropic diffusion model.

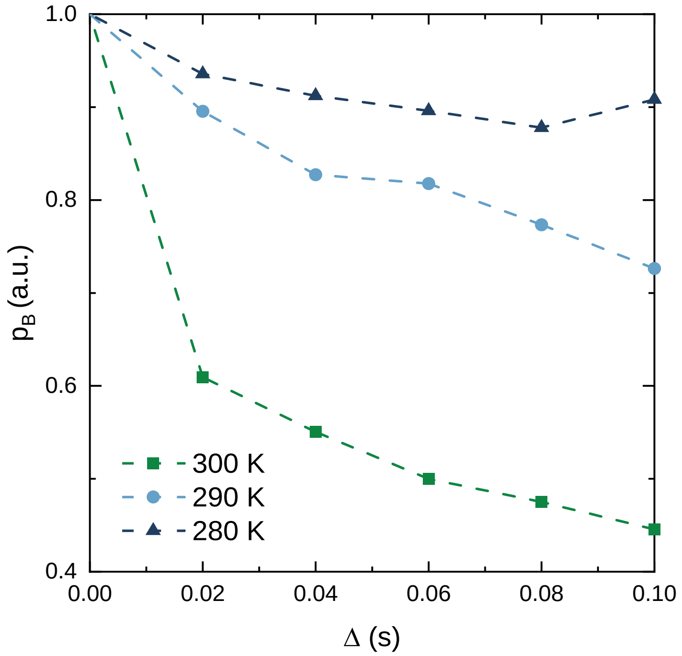

Figure S 23: Population p_B for PI-3-COF-lp as a function of diffusion time. With lower temperature the fraction of molecules diffusing only within the particle during the diffusion time becomes larger, corresponding to a larger population p_B.[29] Dotted lines added to aid visibility.

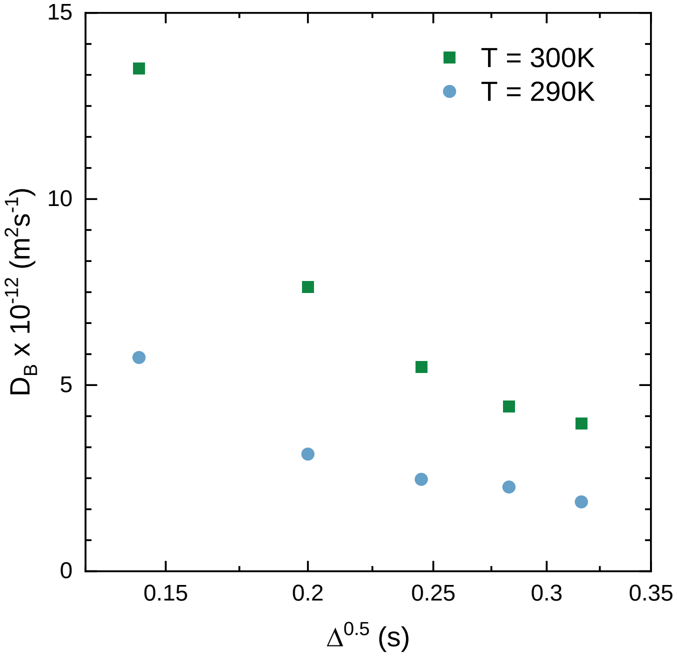

Figure S 24: Diffusion coefficient D_B (intraparticle) of MeCN-loaded PI-3-COF-lp at different diffusion times. The data was plotted against $\Delta^{0.5}$ according to a restricted diffusion model developed for zeolites.[30] As visible from the non-linear trend, this model does not fully reflect the experimental data.

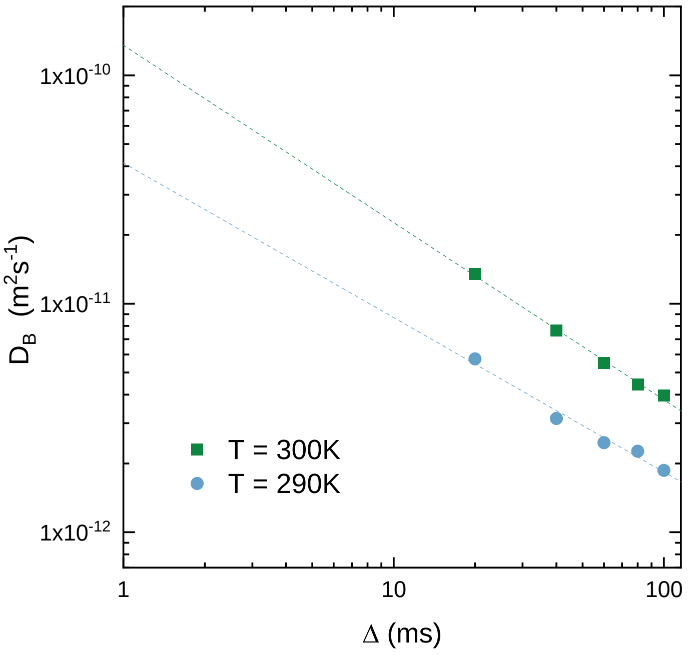

Figure S 25: Diffusion coefficient D_B of MeCN-loaded PI-3-COF-lp at different diffusion times, plotted in a phenomenological log-log presentation. Extrapolation to Δ = 1 ms suggests a short-range diffusion coefficient in the range of $D_B \approx 10^{-10}$ m^2s^{-1} at T = 300 K.[31]

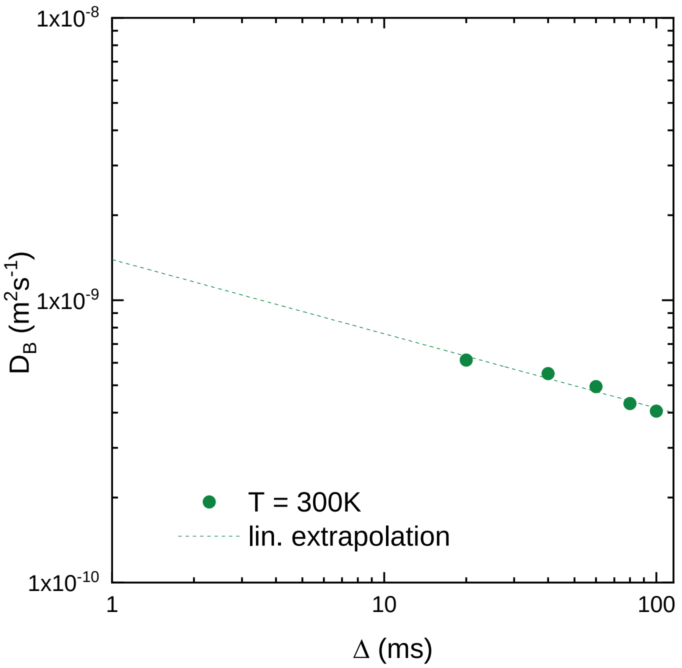

Figure S 26: Diffusion coefficient D_B of MeCN-loaded PI-3-COF-hp at different diffusion times, plotted in a phenomenological log-log presentation. Extrapolation to Δ = 1 ms suggests a short-range diffusion coefficient in the range of $D_B \approx 10^{-9}$ m^2s^{-1} at T = 300 K.[31]

Table S 2: Isotropic diffusion radii ($r = (\langle z^2 \rangle)^{0.5}$) for PI-3-COF-lp and hp for different diffusion times at 300 K for D_B. Notably, the calculated radii neglect diffusion anisotropy and molecular exchange between different regions in the materials.

Δ [ms]	r_{LP} [µm]	r_{HP} [µm]
20	0.73	4.95
40	0.78	6.63
60	0.81	7.71
80	0.84	8.29
100	0.89	8.99

Table S 3: T_1/T_2 relaxation times of other probe molecules imbibed in PI-3-COF.

	PI-3-COF	T [K]	T_1 [s]	T_2 [ms]
chloroform	bulk liquid	300	8.0	1.9 s
chloroform	-lp/-hp	300	2.1/2.0	0.55/0.55
1,4-dioxane	-lp	298	1.2	0.31
cyclohexane	-lp	298	1.4	0.45

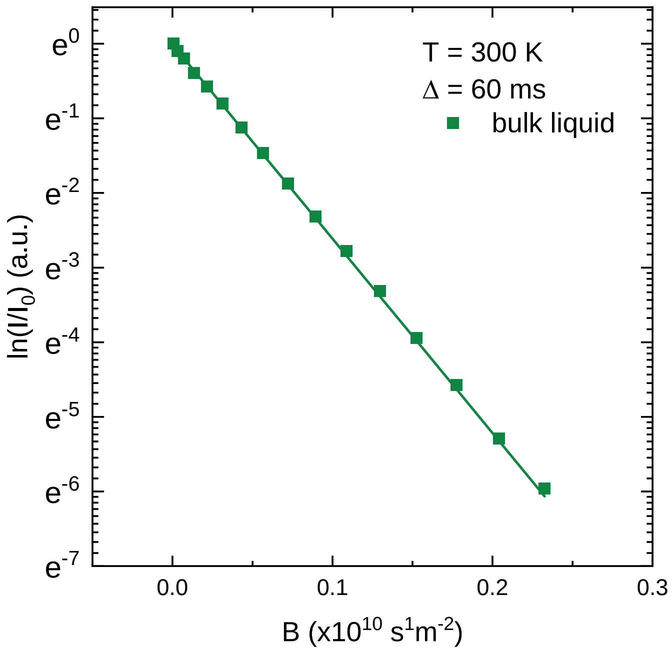

Figure S 27: PFG-NMR spin-echo attenuation for liquid chloroform at T = 300 K. Fitting with a mono-exponential model gives a diffusion coefficient of 2.61(1)x10^{-9} m^2s^{-1}.

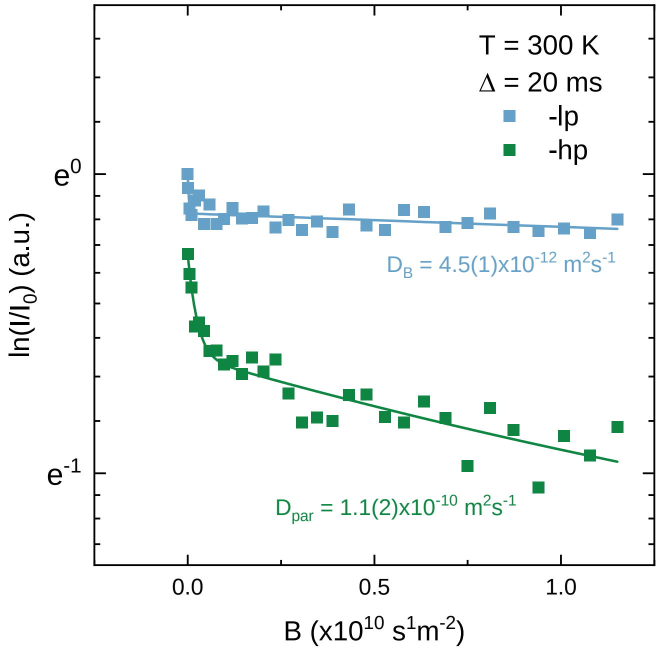

Figure S 28: PFG-NMR spin-echo attenuation for chloroform loaded PI-3-COF samples at loadings of 37 wt% (-lp) and 55 wt% (-hp). Lines represent fits with bi-exponential (-lp) and anisotropic (-hp) models.

References

1. Manz T. A., Limas N. G. Introducing Ddec6 Atomic Population Analysis: Part 1. Charge Partitioning Theory and Methodology. *RSC Adv.* **6**, 47771-47801 (2016).
2. Limas N. G., Manz T. A. Introducing Ddec6 Atomic Population Analysis: Part 2. Computed Results for a Wide Range of Periodic and Nonperiodic Materials. *RSC Adv.* **6**, 45727-45747 (2016).
3. Limas N. G., Manz T. A. Introducing Ddec6 Atomic Population Analysis: Part 4. Efficient Parallel Computation of Net Atomic Charges, Atomic Spin Moments, Bond Orders, and More. *RSC Adv.* **8**, 2678-2707 (2018).
4. Manz T. A. Introducing Ddec6 Atomic Population Analysis: Part 3. Comprehensive Method to Compute Bond Orders. *RSC Adv.* **7**, 45552-45581 (2017).
5. Dubbeldam D., Calero S., Ellis D. E., Snurr R. Q. Raspa: Molecular Simulation Software for Adsorption and Diffusion in Flexible Nanoporous Materials. *Mol. Simul.* **42**, 81-101 (2015).
6. Potoff J. J., Siepmann J. I. Vapor–Liquid Equilibria of Mixtures Containing Alkanes, Carbon Dioxide, and Nitrogen. *AlChE J.* **47**, 1676-1682 (2001).
7. Wick C. D., Stubbs J. M., Rai N., Siepmann J. I. Transferable Potentials for Phase Equilibria. 7. Primary, Secondary, and Tertiary Amines, Nitroalkanes and Nitrobenzene, Nitriles, Amides, Pyridine, and Pyrimidine. *J. Phys. Chem. B* **109**, 18974-18982 (2005).
8. Kamath G., Georgiev G., Potoff J. J. Molecular Modeling of Phase Behavior and

Microstructure of Acetone-Chloroform-Methanol Binary Mixtures. *J. Phys. Chem. B* **109**, 19463-19473 (2005).
9. Mayo S. L., Olafson B. D., Goddard W. A. Dreiding: A Generic Force Field for Molecular Simulations. *J. Phys. Chem.* **94**, 8897-8909 (2002).
10. Lorentz H. A. Ueber Die Anwendung Des Satzes Vom Virial in Der Kinetischen Theorie Der Gase. *Ann. Phys,* **248**, 127-136 (1881).
11. Berthelot D. Sur Le Mélange Des Gaz. *C. R. Chim.* **126**, 15 (1898).
12. Ewald P. P. Die Berechnung Optischer Und Elektrostatischer Gitterpotentiale. *Ann. Phys,* **369**, 253-287 (1921).
13. Kessler C., Schuldt R., Emmerling S., Lotsch B. V., Kästner J., Gross J., Hansen N. Influence of Layer Slipping on Adsorption of Light Gases in Covalent Organic Frameworks: A Combined Experimental and Computational Study. *Microporous Mesoporous Mater.* **336** (2022).
14. Hess B., Kutzner C., van der Spoel D., Lindahl E. Gromacs 4: Algorithms for Highly Efficient, Load-Balanced, and Scalable Molecular Simulation. *J. Chem. Theory Comput.* **4**, 435-447 (2008).
15. Abraham M. J., Murtola T., Schulz R., Páll S., Smith J. C., Hess B., Lindahl E. Gromacs: High Performance Molecular Simulations through Multi-Level Parallelism from Laptops to Supercomputers. *SoftwareX* **1-2**, 19-25 (2015).
16. van der Spoel D., Henschel H., van Maaren P. J., Ghahremanpour M. M., Costa L. T. A Potential for Molecular Simulation of Compounds with Linear Moieties. *J. Chem. Phys.* **153**, 084503 (2020).
17. Nosé S. A Molecular Dynamics Method for Simulations in the Canonical Ensemble. *Mol. Phys.* **52**, 255-268 (2006).
18. Hoover W. G. Canonical Dynamics: Equilibrium Phase-Space Distributions. *Phys. Rev. A: At. Mol. Opt. Phys.* **31**, 1695-1697 (1985).
19. Berendsen H. J. C., Postma J. P. M., van Gunsteren W. F., DiNola A., Haak J. R. Molecular Dynamics with Coupling to an External Bath. *J. Chem. Phys.* **81**, 3684-3690 (1984).
20. Xu H., Cabriolu R., Smit B. Effects of Degrees of Freedom on Calculating Diffusion Properties in Nanoporous Materials. *J. Chem. Theory Comput.* **18**, 2826-2835 (2022).
21. Tanner J. E. Use of the Stimulated Echo in Nmr Diffusion Studies. *J. Chem. Phys.* **52**, 2523-2526 (1970).
22. Sinnaeve D. The Stejskal-Tanner Equation Generalized for Any Gradient Shape-an Overview of Most Pulse Sequences Measuring Free Diffusion. *Concepts Magn. Reson. A: Bridg. Educ. Res.* **40A**, 39-65 (2012).
23. Jerschow A., Müller N. Suppression of Convection Artifacts in Stimulated-Echo Diffusion Experiments. Double-Stimulated-Echo Experiments. *J. Magn. Reson.* **125**, 372-375 (1997).
24. Grunenberg L., Savasci G., Terban M. W., Duppel V., Moudrakovski I., Etter M., Dinnebier R. E., Ochsenfeld C., Lotsch B. V. Amine-Linked Covalent Organic Frameworks as a Platform for Postsynthetic Structure Interconversion and Pore-Wall Modification. *J. Am. Chem. Soc.* **143**, 3430-3438 (2021).
25. Grunenberg L., Savasci G., Emmerling S. T., Heck F., Bette S., Cima Bergesch A., Ochsenfeld C., Lotsch B. V. Postsynthetic Transformation of Imine- into

Nitrone-Linked Covalent Organic Frameworks for Atmospheric Water Harvesting at Decreased Humidity. *J. Am. Chem. Soc.* **145**, 13241-13248 (2023).

26. Keaveney S. T., Schaffarczyk McHale K. S., Stranger J. W., Ganbold B., Price W. S., Harper J. B. Nmr Diffusion Measurements as a Simple Method to Examine Solvent-Solvent and Solvent-Solute Interactions in Mixtures of the Ionic Liquid [Bmim][N(So(2) Cf(3))(2)] and Acetonitrile. *ChemPhysChem* **17**, 3853-3862 (2016).
27. Hurle R. L., Woolf L. A. Self-Diffusion in Liquid Acetonitrile under Pressure. *J. Chem. Soc., Faraday Trans.* **78**, 2233 (1982).
28. Marekha B. A., Kalugin O. N., Bria M., Buchner R., Idrissi A. Translational Diffusion in Mixtures of Imidazolium Ils with Polar Aprotic Molecular Solvents. *J. Phys. Chem. B* **118**, 5509-5517 (2014).
29. Kärger J., Pfeifer H. N.M.R. Self-Diffusion Studies in Zeolite Science and Technology. *Zeolites* **7**, 90-107 (1987).
30. Krutyeva M., Vasenkov S., Yang X., Caro J., Kärger J. Surface Barriers on Nanoporous Particles: A New Method of Their Quantitation by Pfg Nmr. *Microporous Mesoporous Mater.* **104**, 89-96 (2007).
31. Hedin N., Rzepka P., Jasso-Salcedo A. B., Church T. L., Bernin D. Intracrystalline Transport Barriers Affecting the Self-Diffusion of Ch(4) in Zeolites |Na(12)|-a and |Na(12-X)K(X)|-A. *Langmuir* **35**, 12971-12978 (2019).
